# Structural influences on synaptic plasticity: The role of presynaptic connectivity in the emergence of E/I co-tuning

**DOI:** 10.1371/journal.pcbi.1012510

**Published:** 2024-10-31

**Authors:** Emmanouil Giannakakis, Oleg Vinogradov, Victor Buendía, Anna Levina

**Affiliations:** 1 Department of Computer Science, University of Tübingen, Tübingen, Germany; 2 Max Planck Institute for Biological Cybernetics, Tübingen, Germany; Technical University of Munich: Technische Universitat Munchen, GERMANY

## Abstract

Cortical neurons are versatile and efficient coding units that develop strong preferences for specific stimulus characteristics. The sharpness of tuning and coding efficiency is hypothesized to be controlled by delicately balanced excitation and inhibition. These observations suggest a need for detailed co-tuning of excitatory and inhibitory populations. Theoretical studies have demonstrated that a combination of plasticity rules can lead to the emergence of excitation/inhibition (E/I) co-tuning in neurons driven by independent, low-noise signals. However, cortical signals are typically noisy and originate from highly recurrent networks, generating correlations in the inputs. This raises questions about the ability of plasticity mechanisms to self-organize co-tuned connectivity in neurons receiving noisy, correlated inputs. Here, we study the emergence of input selectivity and weight co-tuning in a neuron receiving input from a recurrent network via plastic feedforward connections. We demonstrate that while strong noise levels destroy the emergence of co-tuning in the readout neuron, introducing specific structures in the non-plastic pre-synaptic connectivity can re-establish it by generating a favourable correlation structure in the population activity. We further show that structured recurrent connectivity can impact the statistics in fully plastic recurrent networks, driving the formation of co-tuning in neurons that do not receive direct input from other areas. Our findings indicate that the network dynamics created by simple, biologically plausible structural connectivity patterns can enhance the ability of synaptic plasticity to learn input-output relationships in higher brain areas.

## Author summary

Many studies of learning in biological neural networks have focused on how plausible plasticity rules shape individual connections between neurons in a recurrent network or in feed-forward projections. However, in the latter case, the presynaptic network properties, such as clustered connectivity, strongly influence population dynamics and, thus, the learning process of the projections. Here, we aim to close this gap by showing how non-plastic network structure can strongly influence the outcomes of synaptic learning. We show that unstructured recurrent connectivity and the presence of noise can significantly reduce the ability of synaptic plasticity to separate presynaptic input populations and coordinate excitatory and inhibitory connection development, while the introduction of overlapping clustered structures in fixed or plastic recurrent connectivity can boost synaptic learning. Using Bayesian inference, we identify the optimal connectivity structures for a recurrent network and demonstrate the strong effects that relatively simple connectivity patterns can have on the ability of a network to learn via local plasticity.

## Introduction

Stimulus selectivity, the ability of neurons to respond differently to distinct stimuli, is one of the primary mechanisms for encoding information in the nervous system. This selectivity can range from simple orientation selectivity in lower sensory areas [1–3] to more complex spatiotemporal pattern selectivity in higher areas [4–6]. Such selectivity is shown to be self-organized under the influence of structured input, enabling, for example, the emergence of visual orientation preference in non-visual sensory areas upon rewiring [7] or changing the whiskers representation in the barrel cortex of rats depending on the level of sensory input [8]. The mechanisms underlying the emergence of input selectivity have been the subject of extensive investigation, both through experimental and computational modelling studies, and still remain under active discussion [9–13].

Despite initially attributing stimulus-selectivity to excitatory neurons and their network structure, we now know that inhibitory neurons are also tuned to stimuli, and the coordination of the E/I currents is a central component of efficient neural computation [14, 15]. In particular, it has been shown that excitatory and inhibitory inputs are often correlated [16], with preferred stimuli eliciting stronger excitatory and, with the small delay, stronger inhibitory responses compared to the non-preferred stimuli [17, 18]. This co-tuning of excitation and inhibition is theorized to be beneficial for a variety of computations such as gain control [19, 20], visual surround suppression [21, 22], novelty detection [23] and optimal spike-timing [15, 24].

Although it is still unclear how E/I co-tuning emerges, the dominant view is that it arises via the interaction of several synaptic plasticity mechanisms [25], a hypothesis that has been reinforced by the findings of multiple theoretical studies over the last decade. First, it has been demonstrated that different inhibitory plasticity rules can match static excitatory connectivity [26–29]. More recently, it was also shown that various combinations of plasticity and diverse normalisation mechanisms allow for the simultaneous development of matching excitatory and inhibitory connectivity in feedforward settings [12, 30–32]. Moreover, a variety of plasticity mechanisms has been associated with the formation of stable assemblies [33–37], the creation of E/I balance [38] and the emergence of tuning selectivity [39] in recurrent networks.

However, so far, most of the theoretical studies of synaptic plasticity have focused on identifying the optimal parameters of individual learning rules and normalization mechanisms for specific tasks and ignore the ways in which these mechanisms act within complex network structures that may influence their function. Specifically, biological networks are characterized by highly non-trivial connectivity structures that are known to display varied degrees of clustering and neuron type-specific connectivity patterns [40]. Such network structures give rise to distinct neural dynamics; for example, clustering in recurrent network structure introduces correlations in the activity of similarly tuned neurons [41] and complex interaction between subpopulations of neurons [42]. These types of dynamics fundamentally alter the statistics of population activity that most synaptic plasticity mechanisms rely on for modifying synaptic strength.

Here, we investigate how the development of matching E/I input selectivity in a downstream neuron via synaptic plasticity can be driven by the structure of recurrent connectivity in the input network. We combine excitatory and inhibitory plasticity rules [26, 31, 43] in the feedforward connections of spiking network to develop detailed co-tuning of excitatory and inhibitory connectivity, and we demonstrate that the ability of these plasticity mechanisms to create co-tuning is significantly reduced in the presence of noise and (non-plastic) random recurrent connectivity between the input neurons. We further show that the effects of recurrence and noise on the population activity that drives the formation of matching E/I feedforward weights on a downstream neuron can be fully ameliorated by the introduction of synapse-type specific assemblies of neurons, characterized by local excitation and relatively homogeneous inhibition, an often-observed pattern of cortical connectivity [44, 45]. Our findings demonstrate that network structure can, by influencing population dynamics, significantly modulate the capacity of synaptic plasticity to generate input selectivity in downstream neurons. This highlights a synergistic interaction between structural connectivity and learning mechanisms that can enhance the computational capabilities of brain networks.

## Results

We begin by reproducing previously reported results, validating the emergence of co-tuning in a plastic feedforward network, and introducing measures to capture weight diversity and co-tuning for subsequent analysis. Next, we show that the introduction of strong noise or a random static recurrent connectivity in the presynaptic networks impairs the development of co-tuning by destroying the correlation structure in the activity of the presynaptic population. We then illustrate how specific structures in the static recurrent connectivity can restore the ability of plastic synapses to generate co-tuning. Using analytical results from a reduced linear neural mass model and Bayesian inference for the full network, we identify the optimal connectivity structures in static networks, demonstrating that optimal connectivity is influenced by network sparsity. Finally, we simulate fully plastic networks, confirming that our key observations hold true.

### Co-tuning and its self-organization by synaptic plasticity in a low-noise feedforward setting

**Fig 1:**
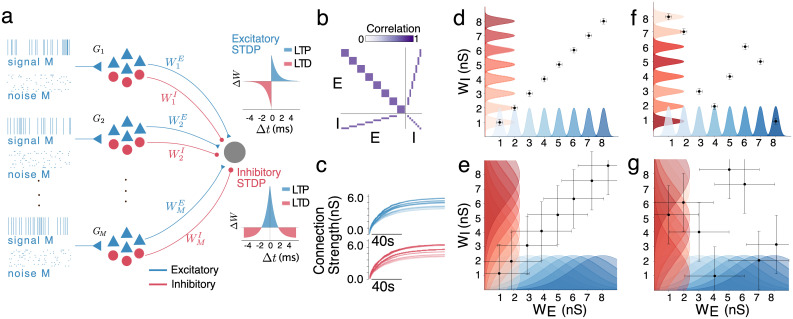
Emergence of co-tuning in a feedforward network. **a.** A diagram of the feedforward network with plastic connections from the different inputs group to the readout neuron.**b.** The correlation matrix of the network’s activity. In the absence of noise, neurons of the same input group are highly correlated. **c.** The development of average E and I weights in an ideal feedforward network with very low noise leads to co-tuned and diverse feedforward connectivity. **d.** An illustration of feedforward connectivity that exhibits both co-tuning and diversity. Different input groups are clearly distinct, and the E and I weights for each group are correlated. The colours of the distribution indicate different groups, and the shading (light to dark) is matched for the corresponding E and I populations; the points and error bars indicate the mean and std of the E and I connectivity of each group. **e.** A co-tuned but not diverse connectivity. E/I weight correlation is maintained, but there is hardly any distinction between groups **f.** A diverse connectivity without E/I weight co-tuning. While each group is distinct from the others, there is no coordination of the E and I connections from the same group **g.** In the absence of weights co-tuning and diversity, the feedforward connectivity lacks any discernible pattern.

We simulate a single postsynaptic read-out unit driven by a population of *N* = 1000 neurons. The pre-synaptic population is divided into *M* groups *G*_*i*_, *i* ∈ {1, …, *M*}. Each group is comprised of *n* = *N*/*M* neurons, of which 80% are excitatory and 20% are inhibitory. These neurons are driven by an identical, group-specific Poisson spike train—a shared external input. Additionally, each neuron receives low-intensity independent external noise [26] that prevents unrealistic total synchrony between the input neurons. This setting, depicted in (Fig 1a), leads to correlated firing among neurons of the same input group (Fig 1b), which is a common setting for studying the effect of different plasticity rules [12, 26, 31].

Input selectivity develops when the post-synaptic neuron responds differently to inputs from different groups (by adjusting its firing rate). This happens when the average excitatory feedforward projections are sufficiently diverse between pre-synaptic groups (Fig 1d and 1f), which leads to groups with stronger feedforward connections eliciting stronger post-synaptic responses upon activation. Moreover, connections from neurons with highly correlated firing (i.e., from the same group) should have a similar strength. To quantify this feature of the network, we define a diversity metric,

$$\begin{array}{c} D=1-\frac{1}{M\cdot \text{Std}(W^{E})}\sum_{i=1}^{M}\;\text{Std}\left(W_{G_{i}}^{E}\right), \end{array}$$ (1)

where $W^{E}\in R^{N}$ is the set of excitatory feedforward connection weights, $W_{G_{i}}^{E}\in R^{N/M}$ is the subset of excitatory feedforward connection weights from input group *i* and Std(⋅) denotes the standard deviation. Diversity *D* ∈ [0, 1] equals unity when the feedforward connections from the same group are the same but differ across groups; *D* is close to zero when the feedforward connections from each group follow the same distribution and different groups cannot be meaningfully distinguished (Fig 1e and 1g).

Brain networks are characterized by a balance of excitatory and inhibitory currents [18, 46, 47]. For a neuron to be balanced, the average inhibitory current must be equal to the average excitatory current during a (relatively short—usually a few mS) time window. Depending on the temporal resolution of this canceling out, the balance can be more “loose” or “tight”, with detailed (“tight”) balance associated with efficient coding [14, 15] and the ability to encode for multiple stimuli [19] (Further discussion in S1 Text Section A). In our specific setting, due to the highly correlated firing in each group, detailed balance can be achieved by matching the relative strengths of the excitatory and inhibitory weights from each group.

To quantify the E/I weight co-tuning, which generates the detailed balance in our simplified network, we use the Pearson correlation coefficient between the mean excitatory and inhibitory weights of each group,

$$\begin{array}{c} CT_{W}=\frac{\text{Cov}(\langle W_{G}^{E}\rangle ,\langle W_{G}^{I}\rangle )}{\text{Std}(\langle W_{G}^{E}\rangle )\cdot \text{Std}(\langle W_{G}^{I}\rangle )}, \end{array}$$ (2)

where $\langle W_{G}^{A}\rangle =(\langle W_{G_{1}}^{A}\rangle ,\langle W_{G_{2}}^{A}\rangle ,\ldots ,\langle W_{G_{m}}^{A}\rangle )$, *A* ∈ {*I*, *E*} and $\langle W_{G_{i}}^{A}\rangle$ is the average projection weight from the excitatory (*A* = *E*) or inhibitory (*A* = *I*) neurons in group *i*, and Cov(*x*_1_, *x*_2_) denotes the covariance of the variables *x*_1_ and *x*_2_. In networks with strong weight co-tuning *CT*_*W*_, the strength of incoming E and I currents is highly correlated (Fig 1d).

We verify that high diversity (*D* ≈ 1) and weight co-tuning (*CT*_*W*_ ≈ 1) can organically emerge via a combination of plasticity mechanisms in the feedforward connections whose trajectories are initialized at the same (small) value. Specifically, the excitatory connections follow the triplet Hebbian STDP rule [48], and the inhibitory connections follow a symmetric rule [26]. We additionally use competitive normalization in both the inhibitory and excitatory connections, which amplifies small transient differences between the firing rates of different input groups and leads to the development of input selectivity [31]. Since neurons in different groups are independent, while neurons in the same group share a strong correlation (Fig 1b), the plasticity protocol generates very strongly correlated E/I weights and strong input selectivity, as shown in (Fig 1c). (For more details on the network’s firing activity, see S1 Text Section C).

Postsynaptic weights with high diversity can be well separated, while those with co-tuned weights produce a match between E and I incoming connections. (Fig 1d) illustrates four possible situations. Observe that it is possible to have weights that are, e.g., diverse (so they can be distinguished) but not co-tuned (so E-I are not correlated) and vice versa: E-I weights are correlated, but weights cannot be separated.

### Noise and recurrent connectivity compromise the ability of STDP to produce E/I co-tuning

Strictly feed-forward networks with relatively low noise levels are unrealistic approximations of complex cortical circuits (which are characterized by noisy inputs and complex recurrent connectivity), and thus their dynamics might deviate significantly from those observed in experiments. Thus, we introduce noise and non-plastic recurrent connectivity in our pre-synaptic network, both ubiquitously present in biological networks [49, 50]. First, we investigate how they individually affect the emerging E/I co-tuning by changing the structure of the correlations between the neurons of the input network. Then, we examine ways in which different connectivity structures can ameliorate these effects.

**Fig 2:**
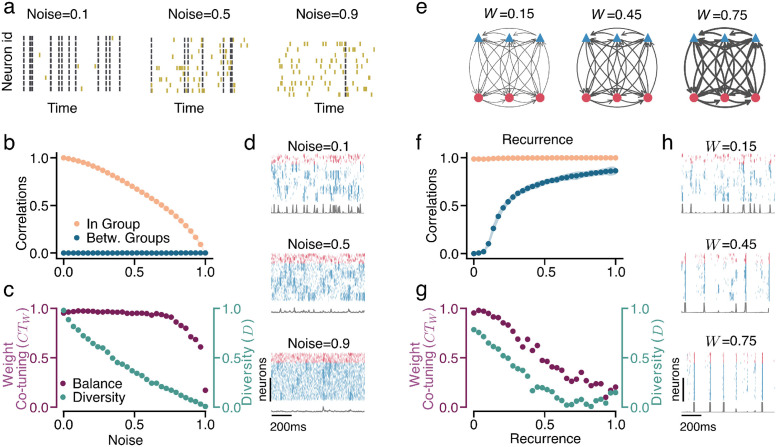
Noise and Recurrence Destroy E/I Co-Tuning. **a.** An illustration of increasing levels of noise in a single input group. In low noise settings, all neurons of the same groups fire at the same time, while as the noise level increases, each neuron fires more and more individual spikes. Joint inputs are shown as black, and individual noise spikes are yellow. **b.** The increase in noise leads to a decrease in in-group correlation (orange), while the between-group correlation (blue) remains low. **c.** An increase in the input noise leads to a reduction in diversity (teal) and, for larger noise intensities, also co-tuning of E/I weights (purple). **d.** Raster plots of input populations’ activity (red, inhibitory neurons; blue, excitatory ones; gray, corresponding firing rate). As the noise increases, the spiking in each group becomes more asynchronous. The traces below (gray line) show the spike count over all neurons in 2ms bins. **e.** An illustration of recurrent connectivity (for three input groups). The coupling parameter *W* controls the mean connection strength. As *W* increases (indicated by thicker connections in the diagram), more of the input a neuron receives comes from other neurons in the recurrently connected network than from the feedforward input. **f.** An increase in the recurrent coupling strength *W* leads to an increase in the between-group correlation (blue), while the in-group correlation (orange) remains high. **g.** The decrease in weight co-tuning and diversity with an increase in the coupling strength. **h.** The spiking activity becomes more synchronous across groups as the coupling strength increases. The traces below (gray line) show the spike count over all neurons in 2ms bins. Simulation parameters not indicated in the text can be found in Tables A, B and C in S1 Text.

We vary the level of noise by changing the fraction of input spikes that are specific to each neuron (noise) vs the shared (signal) input (Fig 2a). This allows us to control the signal-to-noise ratio while keeping the average number of incoming spikes the same. As the noise intensity increases, the cross-correlations within each input group decrease, while the cross-correlations between neurons of different input groups remain very low, (Fig 2b). The effect of this in-group decorrelation is an increased variability in the learned projections to the postsynaptic neuron from neurons of the same input group and, thus, a decrease in the resulting diversity (Fig 2). At the same time, this decorrelation has a much weaker effect on the ability of the plasticity to match E and I feedforward weights from the same groups. This is reflected in the slower reduction of the E/I weight co-tuning, which visibly declines only once the noise becomes overwhelmingly stronger than the input (more than 80% incoming spikes are not shared between neurons of the same group, Fig 2c). Raster plots illustrating the dynamics for different values of noise are shown in (Fig 2d).

Recurrent connectivity in the pre-synaptic network introduces cross-correlations between the neurons from different input groups, which compromises both diversity and E/I weight co-tuning. To test the extent of this effect, we connect the *N* presynaptic neurons (creating a non-plastic recurrent network) with connection probability *p* and use coupling strength *W* (which denotes the mean synaptic strength) as a control parameter. Initially, we only consider fully-connected networks (*p* = 1). By changing *W*, we can control the ratio between the input received from the feedforward connections (whose rate and connection strength are fixed) and the other neurons in the network via recurrent connectivity (Fig 2e). The recurrent connectivity increases cross-correlations between groups while maintaining the high correlation within each input group, (Fig 2f). The effect of these cross-correlations is stronger than the effect of the noise since they affect both the diversity and the weight co-tuning, both of which decline as the recurrent connections become stronger (Fig 2g). As with noise, raster plots for different recurrent connectivity strengths are shown in (Fig 2h).

**Fig 3:**
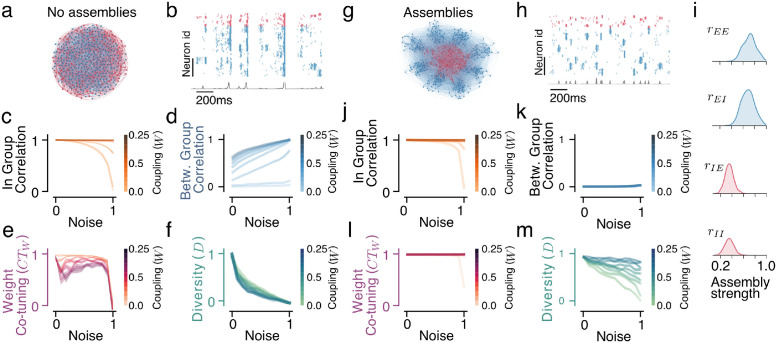
Optimized assemblies of neurons restore the co-tuning in recurrent noisy networks. **a.** Diagram of the network with uniform connectivity **b.** The network activity is characterized by synchronous events across groups. The traces below (gray line) show the spike count over all neurons in 2ms bins. The in-group (**c.**) and between-group (**d.**) correlations for different levels of noise and recurrent connection strength in the uniformly connected network lead to a reduction in the weight co-tuning (**e.**) and weight diversity (**f.**) metrics for different noise and recurrent strength combinations. **g.** Diagram of the network with optimal assembly structure **h.** The network activity becomes more decorrelated across groups. The traces below (gray line) show the spike count over all neurons in 2ms bins. **i.** Approximate posterior distributions of optimal excitatory and inhibitory assemblies strength. The in-group (**j.**) and between-groups (**k.**) correlations for different levels of noise and recurrent connection strength can be almost fully restored by the assembly structure, leading to a restoration of (**l.**) strong weight co-tuning and (**m.**) weight diversity.

The combination of noise and recurrent connectivity affects both in-group and between-group correlations (Fig 3c and 3d), resulting in a reduction of weight co-tuning and diversity as the noise and recurrent connection strength increase, (Fig 3e and 3f). The effects of combined noise and unstructured recurrence are not only a sum of their independent effects, but they can lead to novel effects compounding the impact on the emergence of input selectivity. For example, for strong recurrence (Fig 3d), increasing noise levels seem to lead to an increase of between-group correlations (a counter-intuitive effect, given the decorrelating effects of noise). This is due to the absence of synchronous input to neurons of the same group (due to increased noise, i.e., reduced in-group correlation), which makes synchronisation across groups (and consequently the increase of the between-group correlation) due to recurrent input easier. More details on the effects of noise and recurrence on the network firing are discussed in S1 Text Section C and and D).

We develop a formal description of the effect of noise and recurrence on the correlation structure in a simplified linear neural mass model. To this end, we consider *M* = 8 mesoscopic units instead of the previously studied *M* inter-connected groups, represented by continuous rate variables *x*_*j*_(*t*), *j* = 1, …, *M*. These units evolve in time, subject to stochastic white noise. The linear approximation is justified for any system at a stationary state with a constant average firing rate, and it serves as a simplified model for a wide range of parameters of the spiking network (for details on the linear model and its relation to the spiking network, (see S1 Text Section F and H).

In this simplified case, it is possible to derive analytical equations for all the relevant in- and between-group covariances, which yield the correlation coefficients. These correlations are the solution to a linear system of equations, which can be obtained exactly using numerical methods. Furthermore, one can find close-form solutions in some simple scenarios. For example, in the case of a completely homogeneous network, where all coupling weights are the same, correlation coefficients can be written explicitly (see S1 Text Section F and H). If the coupling strength increases *W* → + ∞, all correlations grow to 1 as $1-O(1/W^{2})$. On the contrary, if we reduce the noise *r* → 0_+_ the correlations will decrease to to $MW/{\left(M-1\right)}^{2}+O\left(r^{-2}\right)$. Both cases eliminate any possible differentiation between the groups, thus compromising the ability of the plasticity mechanisms to create high diversity *D* ≈ 1. Another observation is that in the linear network, increasing noise affects the correlation coefficient quadratically, while coupling increases it linearly. Therefore, since *r* < 1, increasing the coupling has a larger impact on the co-tuning, a consequence that is recovered in the spiking network, consistent with the results shown in (Fig 2b) and (Fig 2e).

### Neuronal type-specific assemblies restore the ability of STDP to produce co-tuning

The homogeneous all-to-all connectivity (Fig 3a and 3b) that we have examined so far is not a realistic assumption and could be particularly detrimental to the self-organization of co-tuning in higher areas. Thus, we examine the impact of different types of inhomogeneous connectivity. In particular, using the idea of functional assemblies (strongly connected neurons that are tuned to the same stimulus [25]), we study whether stronger recurrent connectivity between neurons of the same input group can introduce the necessary correlation structure in the population activity that will allow the plasticity to produce weight diversity and co-tuning.

We maintain the total recurrent input to a neuron constant (the fraction of input coming from the signal/noise vs. the other neurons in the recurrent network, excluding the input from the feedforward connections) while using the ratio of input coming from neurons of the same vs. other input groups as a control parameter. Since we want to vary this ratio independently for each connection type, we define a metric of assembly strength as:

$$\begin{array}{c} r_{ab}=\frac{C_{\text{in}}^{ab}}{C_{\text{in}}^{ab}+C_{\text{out}}^{ab}}=\frac{W_{\text{in}}^{ab}\cdot \bar{r}}{W_{\text{in}}^{ab}\cdot \bar{r}+\left(M-1\right)\cdot W_{\text{out}}^{ab}\cdot \bar{r}}=\frac{W_{\text{in}}^{ab}}{W_{\text{in}}^{ab}+\left(M-1\right)\cdot W_{\text{out}}^{ab}}, \end{array}$$ (3)

where $C_{\text{in}}^{ab}$ in the total recurrent input a neuron of type *b* receives from neurons of type *a*, for *a*, *b* ∈ {*E*, *I*}, of its own input group and $C_{\text{out}}^{ab}$ is the total recurrent input the neuron receives from other input groups, $W_{\text{in}}^{ab}$ the connection strength between neurons of the same group, $W_{\text{out}}^{ab}$ the connection strength between neurons of different groups and $\bar{r}$ is the average firing rate of network neurons (we assume a uniform firing across the network, which can be simplified out of the equation).

We vary assembly strengths for each type of connection *r*_*EE*_, *r*_*EI*_, *r*_*IE*_, and *r*_*II*_ while keeping the total recurrent input to a neuron $C_{\text{in}}^{ab}+C_{\text{out}}^{ab}=\frac{p\cdot N}{M}\cdot \left(W_{\text{in}}^{ab}+\left(M-1\right)\cdot W_{\text{out}}^{ab}\right)=:p\cdot N\cdot W$ constant. Here *W* is the average coupling strength, and *p* is the recurrent connection probability. As in the network without assemblies, for now, we consider fully connected networks (*p* = 1). Thus, we can vary the fraction of input coming to a neuron from its own input group without changing the total recurrent E, or I input it receives.

Since the structure of the feedforward connectivity (diversity and weight co-tuning) that the plasticity converges to is controlled by the correlation structure of the inputs, we can use the correlations as a proxy that is easier to optimize than the connectivity metrics. Specifically, we want to maximize the in-group and minimize the between-group correlations, and we seek the assembly structure that leads to that objective. In the reduced linear neural mass model, we compute the optimal assembly strengths (see S1 Text Section H) analytically and find that strong local excitation and dispersed inhibition restore the desired correlation structure in the network’s activity (see S1 Text Section I). We find that for all combinations of noise and sufficiently strong recurrent connectivity, strong excitatory assemblies (high *r*_*EE*_ and *r*_*EI*_) and uniform inhibitory connectivity (low *r*_*IE*_ and *r*_*II*_) allow the correlating excitatory currents to remain mostly within the input the input group/assembly and maintain high in-group correlation, while the diffused inhibitory currents reduce correlations between groups (for a more detailed discussion of this mechanism see S1 Text Section J). Still, since the reduced model does not account for many essential features of the spiking network, like sparsity of connections, in-group interactions between neurons of the same type, and non-stationary dynamical states of the groups, the analytic solution obtained for the linear neural mass model can serve to develop intuition, but the results need to be validated for the recurrent spiking network.

We now study the effect of various assembly strengths on weight co-tuning and diversity. Instead of directly assessing it, we turn again to the impact of assembly strength on the spiking network’s activity correlation structure. Thus, we search for combinations *r*_*EE*_, *r*_*EI*_, *r*_*IE*_, *r*_*II*_ that lead to the correlation structure (high in-group and low between-group correlations) that is associated with strong E/I weight co-tuning (*CT*_*W*_ ≈ 1) and maximum weight diversity (*D* ≈ 1). To this end, we use sequential Approximate Bayesian Computation (ABC) [51] to minimize a loss function defined to be zero when the in-group correlations are equal one and all between-group correlations vanish (for details, see Methods).

This method allows us to find the approximate posterior distribution of network parameters (the four assembly strengths) that minimize the loss. Afterward, we verify whether connectivity parameters sampled from the approximate posterior lead to the emergence of diversity and co-tuning in the post-synaptic neuron.

Networks with optimized assemblies largely regain the ability to develop E/I co-tuning despite the noise and the non-plastic recurrent connectivity. Assembly strengths that are drawn from the approximate posterior result in a correlation structure very similar to the one observed in a feedforward/low noise network (Fig 3j and 3k), which allows the plasticity to produce a near-optimal structure in the feedforward connections (Fig 3l and 3m). We find that the optimal assembly structure involves very strong *E* ← *E* and *I* ← *E* assemblies and medium-strength *E* ← *I* and *I* ← *I* (Fig 3g and 3i). For details on the impact of assemblies on the network firing and the learned connectivity, see S1 Text Section C and S1 Text Section D.

This connectivity pattern is similar to the optimal pattern of the reduced linear model, albeit with the difference that the reduced model predicted optimal performance for uniform inhibitory weights (i.e., no inhibitory assemblies, *r*_*IE*_ = *r*_*II*_ = 0). This difference can be attributed to the more complex dynamics of the spiking network that require some degree of local inhibition to prevent extreme synchronization (see S1 Text Section G), which can negatively impact the STDP’s ability to produce co-tuning.

This partial specificity of inhibitory recurrent connectivity can be linked to the role of inhibitory tuning in stabilizing network dynamics at the cost of reduced network feature selectivity [52]. In theory, the optimal connectivity pattern in the network would promote competition between different groups, for which completely uniform inhibitory connectivity would be ideal [42]. However, the instability in the population activity induced by such connectivity is detrimental to the emergence of E/I weight co-tuning and input selectivity. Therefore, an intermediate level of specificity in inhibitory recurrent connectivity achieves a balance by maximizing between-group competition (and thus the desired correlation structure) while maintaining stable network dynamics (see S1 Text Section G).

### The sparsity of a network’s recurrent connectivity shifts the optimal assembly structure

Biological neural networks are usually very sparsely connected [53–55], and the sparsity of connections is associated with distinct dynamics [56]. We observed that the impact of noise and recurrence on the deterioration of weight co-tuning and diversity in sparse networks without assemblies is qualitatively similar to fully connected networks. Thus, we examined the ability of neuronal assemblies to produce activity that restores weight co-tuning and diversity in sparsely connected recurrent networks that receive noisy input.

**Fig 4:**
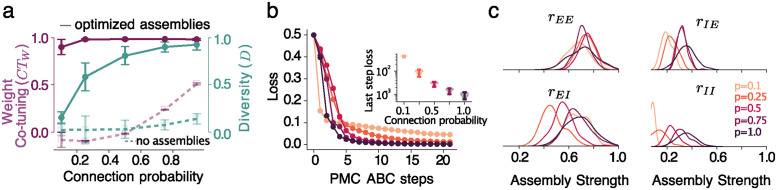
Assemblies improve co-tuning and allow for co-tuning in sparse networks. **a.** weight co-tuning (purple) and diversity (teal) in the networks with assemblies compared to non-structured networks (dashed lines), error bars—standard deviation. The noise level is 0.1 for all sparsities, and the coupling is 1.7 (scaled by 1/*p*). **b.** Loss for the sparser networks is higher, which results in the overall worse performance; the inset shows the loss for 50 accepted samples at the last ABC step. **c.** Posterior distributions of all assembly strengths change with sparsity. Sparser networks require weaker inhibitory assemblies (more uniform connections) to produce co-tuning.

The optimal assembly strength values depend on sparsity levels. We use ABC to discover the approximate posterior distribution of assembly strengths for 5 different levels of sparsity, corresponding to the probability of connection *p* = 1.0, *p* = 0.75, *p* = 0.5, *p* = 0.25, and *p* = 0.1 (Fig 4a). We preserve the total input per neuron across different sparsity levels by scaling the coupling strength inversely proportional to *p*.

As sparsity increases, the ability of assemblies to improve the tuning diminishes. After 21 ABC steps, the overall loss is larger for the sparse networks than for fully connected networks and increases with sparseness, (Fig 4b). Therefore, despite an improvement in the tuning metrics for most sparse networks (compare the dashed and solid lines in (Fig 4a), particularly diversity is strongly affected by sparseness and cannot be recovered by assemblies to the same extent as for the fully connected networks, (Fig 4a). This reduced effectiveness is expected, given the smaller number of connections and the greater variance in the network’s connectivity.

The optimal strength of most assemblies is reduced as the connection probability is decreased (Fig 4c). Specifically, we find that all but *E* ← *E* assemblies should be weaker in sparser networks, with the greatest decrease observed in the *I* ← *I* assemblies that completely disappear for very sparse networks. This could be due to a reduced (compared to fully connected networks) need for within-group recurrent inhibition to prevent completely synchronized behaviours.

### Structured connectivity promotes the emergence of co-tuning in fully plastic recurrent networks

In the previous sections, we analyzed a fixed recurrent presynaptic network that projected onto a single postsynaptic neuron via plastic synapses. While this setup provides valuable insights into how structural features can shape population activity for input selectivity to emerge via STDP, it does not fully capture the dynamics of biological neural networks. In this section, we extend our model to a fully plastic and fully recurrent network, offering a more realistic approximation of the behaviour observed in actual biological systems.

**Fig 5:**
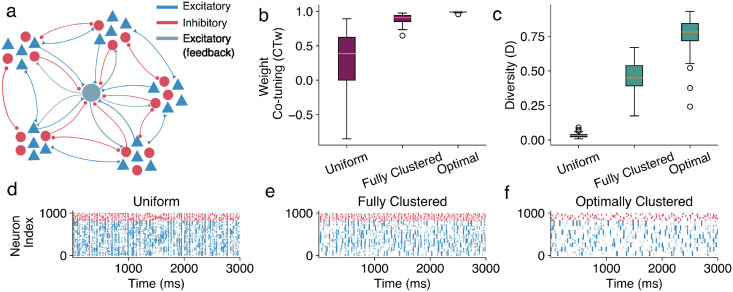
Structured recurrent connectivity can drive synaptic learning even in fully plastic networks. **a.** A diagram of the fully recurrent plastic network. A comparison of the (**b.**) weight co-tuning (*CT*_*w*_) and (**c.**) diversity (*D*) metrics for plastic networks with fully random, fully clustered and optimal connectivity structure. Spike trains of the converged networks with (**d.**) fully random, (**e.**) fully clustered, and (**f.**) optimal recurrent connectivity structures. The activity of different input groups is much more correlated, allowing for easier discrimination in the last network.

In addition to making the recurrent connections between input neurons plastic, we treat the readout neuron as part of the network, and thus, we also introduce sparse feedback connections from the readout neuron to the input neurons (Fig 5a). Since the recurrent connections are now plastic, we cannot implement the assemblies by changing the in-group connection strength. Thus, we use a sparse network (connection probability *p* = 0.25) and control the in-group connection probability. Following the same logic as with the in-group and between-group connection weights (described in Eq 3), we produce in-group and between-group connection probabilities such that the total connection probability (and consequently the total recurrent input) remains constant while the ratio of recurrent input received by a neuron from neurons of its own vs from other groups varies.

We simulate the network with three connectivity structures. A fully random one, where the sparse connections are implemented without any regard for the neurons’ input groups; a fully clustered one, where each input group is almost fully connected, and connections between groups are extremely sparse, and finally, a putative optimally clustered network, which is structured according to the connectivity derived in the previous section for the fixed-weights sparse recurrent network via the Bayesian optimization for the networks of sparsity of *p* = 0.25 (Fig 4c). We measure the co-tuning and weight diversity of the readout neuron, which has now been embedded in the network, projecting randomly with plastic connections to the neurons in the presynaptic network with probability *p*.

The fully random network displays very low co-tuning and diversity; the fully clustered network develops stronger co-tuning and diversity, which are then surpassed by the optimally clustered network (Fig 5b and 5c). Thus, the putatively optimally clustered network outperforms the other two: after the convergence of the plasticity, the population activity becomes much less noisy, and the activity of different groups can be more easily distinguished, which presumably also enables the development of input selectivity in the post-synaptic neuron (Fig 5d, 5e and 5f). The correlation structure of the converged plastic networks verifies this observation, with the putative optimally clustered network having very high in-group and near-zero between-group correlation. This structure is introduced at the beginning of the simulation by the structural connectivity and is maintained throughout the learning process, driving the development of input selectivity in the postsynaptic neuron.

Directly optimizing the fully plastic network is not computationally feasible because, for simulation-based inference, we would need to simulate the network’s activity until all the plastic connections converge in each simulation. However, the connectivity from the static sparse presynaptic network appears to give a good prior for a beneficial connectivity in the fully plastic networks.

Our findings suggest that structured connectivity can drive the statistics of activity even in fully plastic networks and enable the development of specific connectivity in neurons that do not receive direct input from other areas.

## Discussion

Synaptic plasticity is theorized to be responsible for the formation of input selectivity across brain hierarchies, including in brain areas that only receive input from highly recurrent networks. Here, we demonstrated how the structure of non-plastic presynaptic recurrent connectivity could hinder or boost the ability of synaptic plasticity mechanisms [12, 26, 27, 31] to generate input selectivity in neurons of higher areas. We find that strong excitatory connectivity among neurons tuned to the same input, combined with broader inhibition, creates population activity with a beneficial statistical structure that enables the formation of co-tuned projections by plasticity, potentially fostering input selectivity.

How different plasticity mechanisms shape neural connectivity, such as the formation of E/I co-tuning [12, 26, 27, 31] in feedforward networks or neural assemblies in recurrent networks [26, 34–36], has been a topic of extensive theoretical research. Nevertheless, the opposite effect -the ways in which fixed connectivity can shape the effects of synaptic plasticity—has only been studied in very specialized cases [57]. This omission partially obscures the two-way interaction between connectivity and synaptic plasticity in biological neural networks. While synaptic plasticity constantly modifies some aspects of neural connectivity, it acts within the many constraints of network structure that are either constant throughout an organism’s lifetime or change via structural adaptation mechanisms that act on timescales slower than synaptic plasticity [58, 59]. Effectively, this means that synaptic plasticity relies on population activity originating from networks with highly intricate connectivity structures very different from those of random networks [33, 60]. Given that most synaptic plasticity mechanisms fundamentally depend on the statistics imposed by network activity, it is reasonable to assume that the network structure highly impacts the behaviour of synaptic plasticity.

Cortical connectivity is known to be highly clustered [61], and the clustering has functional as well as spatial determinants. For example, neurons that share common inputs [62] or targets [63] are more likely to form recurrent connections between themselves [64, 65]. Moreover, excitatory cells with similar receptive fields are known to form strong reciprocal connections [66], which determine neural responses. Additionally, cortical networks have been shown to present specific correlation structures early in development [67], suggesting that recurrent cortical connectivity is at least partially structured before sensory inputs are present.

Clustered networks display distinct dynamics, including competition between clusters [42, 68] and slower timescales [41, 69], both of which can be useful for computations. Additionally, there is strong evidence that groups of highly interconnected neurons (neuronal assemblies) share common functions within recurrent networks [37, 70, 71]. Moreover, evidence has accumulated [72, 73] that different neuron types (excitatory and inhibitory subtypes) follow distinct spatial connectivity patterns, which have implications for neural computation [74]. Thus, our findings complement the ongoing research on computational implications of recurrent neural connectivity in biological networks by suggesting a link between specific, fixed connectivity patterns and local learning in feedforward projections to downstream populations via synaptic plasticity.

While the connectivity pattern we identify in our study is biologically plausible, the extent to which it is realized *in vivo* remains unclear. Further experimental studies in the connectivity patterns of different neuron types are necessary to model different network connectivities and study their dynamics effectively. On a theoretical side, more research is needed to uncover whether the synapse-type specific assembly structures we identify can emerge in plastic networks without any prior structure. Specifically, while there have been several studies investigating the emergence of stable assemblies via STDP [33–35, 75], the protocols by which assemblies of different pre-selected strengths for each connection type could arise remain unclear. Potential mechanisms include structural plasticity and variation in the learning rates of different synaptic types. Additionally, the presence of different regulatory interneurons, which have already been studied in the context of assembly formation [34], could play a role in modulating the relative assembly strengths of different connections.

For our study, we parameterized the network connectivity by adopting a quantitative metric for the strength of different types of neuronal assemblies. This resulted in a low-dimensional parameter space and allowed us to use rejection sampling-based ABC [51] to infer the optimal assembly strengths. One limitation of this technique is that it suffers from the curse of dimensionality and typically requires a large simulation budget [76, 77]. Therefore, extensions of the current work using higher dimensional connectivity parameters or simultaneous inference of the connectivity and neuron parameters will require more efficient simulation-based methods such as neural posterior estimation [78]. Alternatively, direct optimization of each recurrent weight via gradient-based methods [79, 80] may uncover more intricate connectivity patterns that are not limited to the specific network parametrization we chose.

To summarize, we identified how particular presynaptic connectivity structures could be a favourable or detrimental substrate for plasticity to develop co-tuning of excitation and inhibition on neuronal projections. Our study is the first step in illuminating the two-way dependence between the non-plastic structural features of a network’s connectivity and synaptic plasticity, which can motivate further research on this intricate interaction.

## Materials and methods

### Neuron model

We modelled all neurons of our networks as leaky integrate-and-fire (LIF) neurons with leaky synapses [81]. The evolution of their membrane potential is given by the ODE:

$$\begin{array}{c} C_{m}\cdot \frac{dV(t)}{dt}=g_{\text{leak}}\cdot \left(V_{\text{rest}}-V\left(t\right)\right)+g_{I}\left(t\right)\cdot \left(V_{I}-V\left(t\right)\right)+g_{E}\left(t\right)\cdot \left(V_{E}-V\left(t\right)\right), \end{array}$$ (4)

where *V*_rest_ is the neuron’s resting potential, *V*_*E*_, *V*_*I*_ are the excitatory and inhibitory reversal potentials and *g*_leak_ the leak conductance. Additionally, the excitatory and inhibitory conductances *g*_*E*_, *g*_*I*_ decay exponentially over time and get boosts upon excitatory or inhibitory pre-synaptic spiking, respectively, as

$$\begin{array}{c} \begin{array}{cc} \frac{dg_{E}\left(t\right)}{dt} & =-\frac{g_{E}\left(t\right)}{\tau_{E}}+\bar{g_{E}}\cdot \sum_{j}W_{j}^{E}\cdot \sum_{f}\delta \left(t-t_{j}^{f}\right),\\ \frac{dg_{I}\left(t\right)}{dt} & =-\frac{g_{I}\left(t\right)}{\tau_{I}}+\bar{g_{I}}\cdot \sum_{j}W_{j}^{I}\cdot \sum_{f}\delta \left(t-t_{j}^{f}\right). \end{array} \end{array}$$ (5)

Here $t_{j}^{f}$ denotes the time at which the *f*-th spike of the *j* − th neuron happened and *δ*(*t*) is the Dirac’s delta function. When the membrane potential reaches the spiking threshold *V*_th_, a spike is emitted, and the potential is changed to a reset potential *V*_reset_. Finally, the neurons have an absolute refractory period (during which no spikes are emitted even when the spiking threshold is reached) between spikes of *τ*_ref_ = 5*ms*.

We would like to remark that both $W_{i}^{E},W_{i}^{I}>0$, since the effect of inhibition is encoded on Eq (4). However, for illustration purposes inhibitory weights are currents are shown to be negative. For instance, this happens in (Fig 1b). An alternative neuron model is discussed in S1 Text Section B.

### Network input

The external input to each of the 1000 pre-synaptic neurons is the mixture of two Poisson spike trains. The first Poisson spike train is shared with all the other neurons of the same group, while the second Poisson spike train is the individual noise of the neuron,

$$\begin{array}{c} C_{\text{total}}=C_{\text{signal}}+C_{\text{noise}}, \end{array}$$ (6)

where *C*_signal_ ∼ Poisson((1 − *c*) ⋅ *f*_0_) and *C*_noise_ ∼ Poisson(*c* ⋅ *f*_0_). Here, *f*_0_ is the total firing rate of the input, and *c* is the strength of the noise. *C*_signal_ is the same for all neurons of the same input group, while *C*_noise_ is individual to each neuron.

### Recurrent connectivity

The recurrent connectivity is implemented in two different versions for the fixed and plastic versions.

#### Non-plastic recurrent connectivity

The non-plastic recurrent connectivity between the input neurons is defined by a coupling strength parameter *W*, which defines the average synaptic strength and a connection probability *p*, which defines the sparsity. The connectivity is implemented as follows:

At first, an adjacency matrix *A* is defined, which implements an Erdős–Rényi connectivity with connection probability *p* (i.e., a connection between any two neurons is implemented with connectivity *p*, independently of any other connection). Then, using the coupling strength parameter *W*, and the given assembly strength for each connection type *r*_*EE*_, *r*_*EI*_, *r*_*IE*_, and *r*_*II*_, we extract the parameters $W_{\text{in}}^{ab}$ and $W_{\text{out}}^{ab}$ for each connection type (*a*, *b* ∈ {*E*, *I*}) according to Eq 3. The inhibitory weights $W_{\text{in}}^{IE},W_{\text{out}}^{IE},W_{\text{in}}^{II}$ and $W_{\text{out}}^{II}$ are scaled by a parameter *g*_*s*_ which is set to counterbalance the slower inhibitory synapse dynamics and the smaller number of *I* neurons. This scaling leads to an approximately balanced network across implementations. Once the connectivity strengths are calculated, for each pre and post-synaptic neuron pair *i* and *j*, we set the connection between them as

$$\begin{array}{c} w_{ij}=\{\begin{array}{c} 0,\;\text{if}\;A_{ij}=0\\ \alpha_{ij}∼\left|N\left(W^{ij},c\cdot W^{ij}\right)\right|,\;\text{if}\;A_{ij}=1 \end{array} \end{array}$$ (7)

where *W*^*ij*^ is the appropriate connectivity strength ($W_{\text{in}}^{ab}$, $W_{\text{out}}^{ab}$ for *a*, *b* ∈ {*E*, *I*}) depending on the neuron type of neurons *i* and *j* and whether they belong to same assembly or not. The parameter *c*, which scales the standard deviation, was normally set to 0.1, but we also examined narrower and broader distributions with similar results.

We finally considered an alternative, log-normal distribution of weights, which increased variability but largely lead to the same results.

#### Plastic recurrent connectivity

In the plastic recurrent network case, instead of varying the connection strengths for in-group and between-group connections, we use the total connection probability *p* and given assembly strength for each connection type *r*_*EE*_, *r*_*EI*_, *r*_*IE*_, and *r*_*II*_, we extract parameters $p_{\text{in}}^{ab}$ and $p_{\text{out}}^{ab}$ for each connection type (*a*, *b* ∈ {*E*, *I*}) according to Eq 3. These parameters give the probability that a connection of a specific type is implemented in the recurrent network, which creates an inhomogeneous adjacency matrix *A* that implements the different levels of clustering for each connection type.

Once the adjacency matrix has been defined, we set the initial connection strength for pre and post-synaptic neuron pair *i* and *j* as:

$$\begin{array}{c} w_{ij}=\{\begin{array}{c} 0,\;\text{if}\;A_{ij}=0\\ \alpha_{ij}∼\left|N\left(W,c\cdot W\right)\right|,\;\text{if}\;A_{ij}=1 \end{array} \end{array}$$ (8)

where *W* is the coupling strength parameter, which we scale for inhibitory connections, similarly to the non-plastic network. The resulting connectivity reflects the initial conditions for the plastic recurrent network and the non-zero connections are updated according to the same plasticity protocol that is used to learn the feedforward connectivity in the networks with fixed recurrent connectivity.

### Plasticity

#### Triplet excitatory STDP

The excitatory connections are modified according to a simplified form of the triplet STDP rule [43], which has been shown to generalize the Bienenstock–Cooper–Munro (BCM) rule [9] for higher-order correlations [48]. In our implementation of the triplet rule, the firing rates of the pre-synaptic excitatory neurons and the post-synaptic neuron are approximated by traces with two different timescales (we use the same timescales for the fast and slow traces of the pre and postsynaptic neuron):

$$\begin{array}{c} \frac{dy_{k}^{E}\left(t\right)}{dt}=-\frac{y_{k}^{E}\left(t\right)}{\tau_{1}^{estdp}}+\sum_{f}\delta \left(t-t_{k}^{f}\right), \end{array}$$ (9a)

$$\begin{array}{c} \frac{dz_{k}^{E}\left(t\right)}{dt}=-\frac{z_{k}^{E}\left(t\right)}{\tau_{2}^{estdp}}+\sum_{f}\delta \left(t-t_{k}^{f}\right), \end{array}$$ (9b)

$$\begin{array}{c} \frac{dx_{1}\left(t\right)}{dt}=-\frac{x_{1}\left(t\right)}{\tau_{1}^{estdp}}+\sum_{f}\delta \left(t-t_{x}^{f}\right), \end{array}$$ (9c)

$$\begin{array}{c} \frac{dx_{2}\left(t\right)}{dt}=-\frac{x_{2}\left(t\right)}{\tau_{2}^{estdp}}+\sum_{f}\delta \left(t-t_{x}^{f}\right), \end{array}$$ (9d)

where $\tau_{1}^{estdp}<\tau_{2}^{estdp}$ are the two timescales of the plasticity rule, $y_{k}^{E}\left(t\right),z_{k}^{E}\left(t\right)$ and *x*_1_(*t*), *x*_2_(*t*) represent the slow and fast traces of the *k*-th excitatory pre-synaptic and the single post-synaptic neuron respectively while $t_{k}^{f}$ and $t_{x}^{f}$ are their respective firing times The function *δ*(*x*) represents a Dirac’s delta. The connection weights are updated upon pre and post-synaptic spiking according to

$$\begin{array}{c} \Delta W_{k}^{E}=\eta_{E}\cdot A_{LTP}\cdot x_{2}\left(t\right)\cdot y_{k}^{E}\left(t\right)\cdot \sum_{f}\;R\left(t-t_{x}^{f}\right)-\eta_{E}\cdot A_{LTD}\cdot x_{1}\left(t\right)\cdot z_{k}^{E}\left(t\right)\cdot \sum_{f}\;R\left(t-t_{k}^{f}\right), \end{array}$$ (10)

where *η*_*E*_ is the excitatory learning rate and *A*_*LTP*_, *A*_*LTD*_ the amplitudes of long term depression and potentiation respectively. Despite scaling down the LTD amplitude to account for higher presynaptic firing rates, the rule remains slightly LTD dominated in our experiments, a setting that has been observed in experimental studies [82, 83]. The function *R*(*x*) is defined as:

$$\begin{array}{c} R\left(x\right)=\{\begin{array}{cc} 1, & x=0\\ 0, & x\neq 0 \end{array} \end{array}$$ (11)

In numerical simulation, if spikes have been produced in the last few timesteps, so in practice *R*(*t*) = 1 for *t* ∈ [−*δt*, 0] for a small *δt*. Hence, *R*(*t*) is a rectangular function. Different parameters and learning rules for the excitatory plasticity are discussed in (S1 Text Section E.i and E.ii).

#### Inhibitory STDP

We used the learning rule first proposed in [26] for the inhibitory connections. Approximations of the firing rates are kept via a trace for each of the pre-synaptic inhibitory neurons as well as the post-synaptic neuron,

$$\begin{array}{c} \frac{dy_{k}^{I}}{dt}=-\frac{y_{k}^{I}}{\tau^{istdp}}+\sum_{f}\delta \left(t-t_{k}^{f}\right), \end{array}$$ (12a)

$$\begin{array}{c} \frac{dx}{dt}=-\frac{x}{\tau^{istdp}}+\sum_{f}\delta \left(t-t_{x}^{f}\right), \end{array}$$ (12b)

where *τ*^*istdp*^ is the single timescale of the plasticity rule, $y_{k}^{I}$ and *x* are the traces of the the *k*_*th*_ inhibitory pre-synaptic and the single post-synaptic neuron, and $t_{k}^{f},\;t_{x}^{f}$ are their respective spike times. The connection weights are updated upon pre and post-synaptic spiking as

$$\begin{array}{c} \Delta W_{k}^{I}=\eta_{I}\cdot \left(x\left(t\right)-2\rho_{0}\tau^{istdp}\right)\cdot \sum_{f}R\left(t-t_{k}^{f}\right)+\eta_{I}\cdot y_{k}^{I}\left(t\right)\cdot \sum_{f}R\left(t-t_{x}^{f}\right). \end{array}$$ (13)

Here, *η*_*I*_ is the inhibitory learning rate, and *ρ*_0_ is the target firing rate of the post-synaptic neuron. The rectangular function *R*(*t*) is defined in Eq (10).

#### Weight normalization

Due to the instability of the triplet STDP rule, some normalization mechanism is needed to constrain weight development. We use a modified version of the competitive normalization protocol proposed in [31], which we adapt for spiking neurons.

Specifically, we normalize the *k*-th connection every time there is a weight update (i.e., upon pre or postsynaptic spiking):

$$\begin{array}{c} W_{k}^{A}\left(t\right)\leftarrow (1-\eta_{N})\cdot W_{k}^{A}\left(t\right)+\eta_{N}\cdot W_{k}^{A}\left(t\right)\cdot \frac{W_{target}^{A}}{\sum_{i=1}^{N_{A}}W_{i}^{A}\left(t\right)},\;A\in \left\{E,I\right\}. \end{array}$$ (14)

Where $W_{target}^{A}$ is the target total weight of each connection type and *η*_*N*_ is the normalization rate. In the recurrent plastic networks, the $W_{target}^{A}$ for the recurrent neurons is determined by the coupling strength *W*. The normalization pushes the sum of the excitatory and the sum of the inhibitory feedforward connections weights close to the set target total weights $W_{target}^{E}$ and $W_{target}^{I}$ over time. The implications of implementing a regular normalization step (on every time step only when spiking occurs) are discussed in S1 Text Section E.iv.1. Moreover, the implications of using different normalization rates *η*_*N*_ are discussed in S1 Text Section E.iv.2.

An alternative way to stabilize the weights via subtractive normalization of only the excitatory synapses [12, 23, 33] was also considered leading to comparable results (see S1 Text Section E.v).

### Approximating the posterior distribution of the model parameters

To estimate the set of parameters that lead to high in-group correlations and low out-group correlations, we used simulation-based inference [76]. The basic idea is to use simulation with known parameters to approximate the full posterior distributions for the model given the required output, i.e., the distribution of parameters and samples from which produce the required correlation structure. We use sequential Approximate Bayesian Computation (ABC) [51] to approximate the posterior distribution. We define a loss function that maximizes in-group correlations and minimizes between-group correlations:

$$\begin{array}{c} L=-\alpha C_{in}^{2}-\beta [{\left(1-C_{out}^{EE}\right)}^{2}+{\left(1-C_{out}^{EI}\right)}^{2}+{\left(1-C_{out}^{II}\right)}^{2}] \end{array}$$ (15)

We define a uniform prior *p*(*θ*). A set of parameters *θ* = [*r*_*ee*_, *r*_*ei*_, *r*_*ie*_, *r*_*ii*_] is sampled from it and used to run the simulations for 3 seconds. From the simulation results, correlations are computed, which allows us to obtain the loss. We accept a parameter set if the loss is below the error *ϵ*, and keep sampling until the number of accepted samples is 60. We use the kernel density estimate on the accepted samples to obtain an approximate posterior. Next, we rescale this approximate posterior with the original prior to obtain a proposal distribution that we use as a prior in the next step of the ABC. In each step, we reduce *ϵ* by setting it to the 75th percentile of the losses for the accepted samples (see [51] for more details). As a rule, we run 20 to 30 steps of the sequential ABC until the loss converges. We run separate fits for networks with different levels of sparsity with connection probabilities *p* = 0.1, 0.25, 0.5, 0.75, 1.0. The fitting was done using a modified version of the simple-abc toolbox https://github.com/rcmorehead/simpleabc/ for python.

### Reduced model

The dynamics of the system can be studied analytically using a simplified, reduced linear model. Here, each pair of variables (*x*_*i*_, *y*_*i*_) represents the excitatory and inhibitory mean firing rate of a neuron group. In theory, these variables display complicated non-linear interactions that arise from the microscopic details of the LIF spiking network and synapse dynamics. However, in the stationary state –and away from any critical point– a linearised model can capture the essential features of the correlations between different populations.

Internal noise, modelled as independent Poisson trains to each individual neuron, becomes Gaussian white noise in the large-population limit, characterized by zero mean and variance *σ*_int_. Each population is affected by different internal fluctuations. For simplicity, external noise, which is applied as the same train of Poisson spikes to all the neurons inside an input group, will also be approximated as a Gaussian white noise of mean *η*_0_ and variance *σ*_ext_.

Therefore, the simplified linear model reads:

$$\begin{array}{c} {\dot{x}}_{i}=ax_{i}+by_{i}+\frac{1}{M-1}\sum_{j\neq i}(W^{E\leftarrow E}x_{j}+W^{E\leftarrow I}y_{j})+\sigma_{\text{int}}\xi_{i}^{x}(t)+\sigma_{\text{ext}}\eta_{i}(t)+\eta_{0}, \end{array}$$ (16a)

$$\begin{array}{c} {\dot{y}}_{i}=cx_{i}+dy_{i}+\frac{1}{M-1}\sum_{j\neq i}(W^{I\leftarrow E}x_{j}+W^{I\leftarrow I}y_{j})+\sigma_{\text{int}}\xi_{i}^{y}(t)+\sigma_{\text{ext}}\eta_{i}(t)+\eta_{0}, \end{array}$$ (16b)

where *M* is the number of populations, *a*, *b*, *c*, *d* are parameters controlling in-group recurrent coupling, and *W*^*E* ← *E*^, *W*^*E* ← *I*^, *W*^*I* ← *E*^, *W*^*I* ← *I*^ are couplings between different clusters. Internal noise for each population is represented by $\xi_{i}^{x,y}\left(t\right)$, while external noise is notated as *η*_*i*_(*t*). All noises are uncorrelated, meaning that

$$\begin{array}{c} \langle \xi_{i}^{c}\xi_{j}^{c^{\prime }}\rangle =\delta_{cc^{\prime }}\delta_{ij}\delta (t-t^{\prime }), \end{array}$$ (17a)

$$\begin{array}{c} \langle \xi_{i}^{c}(t)\eta_{j}(t^{\prime })\rangle =0\;\forall i,j,t,t^{\prime }, \end{array}$$ (17b)

$$\begin{array}{c} \langle \eta_{i}(t)\eta_{j}(t^{\prime })\rangle =\delta_{ij}\delta (t-t^{\prime }), \end{array}$$ (17c)

with *c*, *c*′ = {*x*, *y*}, and where 〈…〉 represents an ensemble average, i.e., an average over noise realizations. From this model, it is possible to obtain closed equations for Pearson correlation coefficients (see S1 Text Section H for details). Notice that stochastic differential equations are never complete without an interpretation, and we choose to interpret these in the Itô sense, which will be relevant for computations. Tables of all the parameters used in our simulations are given in S1 Text Section K Tables A, B and C.

## Supporting information

S1 Text Supplementary information. (PDF)
