# Supplementary material for: Structural influences on synaptic plasticity: The role of presynaptic connectivity in the emergence of E/I co-tuning
Source: PLoS Comput Biol. 2024 Oct 31;20(10):e1012510. doi: 10.1371/journal.pcbi.1012510 (PMC11556753; doi:10.1371/journal.pcbi.1012510)
Supplement: S1 Text — (PDF) [file pcbi.1012510.s001.pdf]

Supplementary information
Structural influences on synaptic plasticity: the role of presynaptic connectivity in the emergence of E/I co-tuning

Emmanouil Giannakakis, Oleg Vinogradov, Victor Buendía, Anna Levina

Contents

A. E/I co-tuning and input selectivity 1

B. The effects of inhomogeneous connectivity on the population activity are independent of the neuron model 2

C. Firing behaviour of the presynaptic network 2

D. Learned connectivity and the dynamics of the post-synaptic neuron 4

E. The effects of inhomogeneous connectivity are independent of the plasticity protocols' details 5
- i. Excitatory plasticity: Parameters of the Triplet rule 5
- ii. Excitatory plasticity: Triplet vs Pair rule 7
- iii. Inhibitory learning rule target rate 7
- iv. Alternative implementations of the competitive Normalization 8
  - 1. Regular vs event-based normalization steps 8
  - 2. "Soft" vs. "Strict" normalization: The impact of the normalization rate 9
- v. Subtractive Normalization with modified input 9

F. Convergence of weights to an eigenvector of a modified covariance matrix under plasticity 10

G. Perturbation of the optimal assembly strengths leads to diverse effects on the network's activity 11

H. Reduced model calculations 12
- i. Derivation of the equations 12
- ii. Solutions for the homogeneous network 14

I. Clustering optimisation 14

J. The inferred connectivity structure encourages competition between assemblies 16

K. Tables of parameters 17

References 18

A. E/I co-tuning and input selectivity

In our study, we use the weight diversity as a proxy for input selectivity. Verify that this mapping is reasonable even for networks with very detailed E/I balance. Specifically, we model 2 networks with fixed, perfectly balanced connectivity. The first of the two has tuned excitation (Fig Aa) and totally flat inhibition (leading to loose balance), while the second has near-perfect co-tuning of its incoming E and I incoming weights, which leads to tight balance (Fig Ab).

We sequentially activate different input groups and record the post-synaptic neuron's response in terms of incoming currents and firing rate. We see that while there is a small difference between the incoming currents in the two cases (Fig Ab and d), the firing rate of the postsynaptic neuron is significantly different between the two networks (Fig Ae).

In particular, we see that compared to the network with flat inhibition, the responses of the network with the co-tuned E/I weights are significantly reduced but still clearly different for different inputs, allowing discrimination. This is consistent with recent theoretical findings [1] about inhibitory tuning reducing input selectivity.

However, the co-tuned network displays a reduced firing rate, which suggests the possibility for more efficient encoding as well as a partial encoding for non-preferred inputs (i.e., a small but detectable response for the input group associated with small input weights) in contrast to the neuron with flat inhibition, which is fully silent for the non-preferred inputs (i.e., those for which it receives stronger inhibition than excitation). This agrees with earlier findings on the benefits of weights co-tuning in terms of efficiency and coding [2].

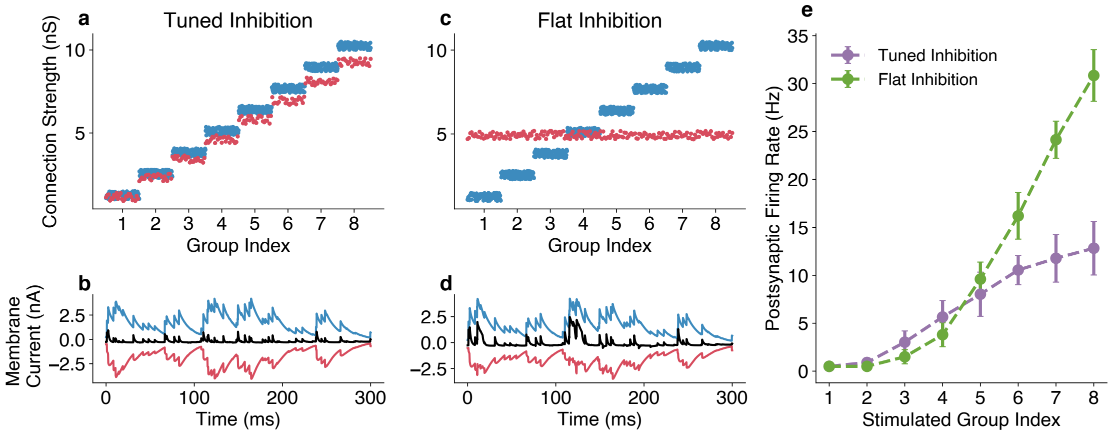

FIG. A. **E/I Weights and input selectivity: a.** The feedforward connectivity and **b.** the resulting postsynaptic currents for a network with flat co-tuned E/I feedforward weights feedforward inhibition. **c.** The feedforward connectivity and **d.** the resulting postsynaptic currents for a network with flat feedforward inhibition. **e.** The firing of the postsynaptic neuron upon activation of different input groups for each network. Flat inhibition leads to sharper responses while co-tuning leads to lower FR and a more gradual increase of activity for different input activation.

This suggests that while the detailed co-tuning of feedforward E/I weights might lead to a reduction in the sharpness of the post-synaptic neuron's tuning, it still enables discrimination between inputs and, indeed, can encode for a broader range of inputs (responses even for non-preferred stimuli) in a more energetically efficient way (lower firing rate).

B. The effects of inhomogeneous connectivity on the population activity are independent of the neuron model

In order to ensure that our results are independent of the exact neuron model we are using, we repeated the bayesian fitting with a simplified network of LIF neurons with current based synapses.

Specifically, the voltage is given by:

$$\frac{dV(t)}{dt} = (V_{\text{rest}} - V(t))/\tau + \sum_j W_j^E \cdot \sum_f \delta(t - t_j^f) - \sum_j W_j^I \cdot \sum_f \delta(t - t_j^f), \tag{1}$$

where V_{rest} is the neuron's resting potential and τ is the membrane timescale. Here t_j^f denotes the time at which the f-th spike of the $j-$th neuron happened. When the membrane potential reaches the spiking threshold V_{th}, a spike is emitted, and the potential is changed to a reset potential V_{reset}.

We repeat the fitting with ABC for a network with this model, using the same objective function (maximizing in-group and minimizing between-group correlations) and we find that the optimal assembly strength distribution for all four connection types we identified for the original network remains very similar to the ones we identified for the original model.

C. Firing behaviour of the presynaptic network

In order to better understand the effects that the introduction of noise and non-plastic recurrent connections has on the population activity of the presynaptic network, we calculate a number of metrics that quantify population activity for different connectivity structures.

At first, we look at a feedforward network with relatively low noise (noise intensity is set to 0.15). This setting produces activity that is ideal for the plasticity to produce diverse and co-tuned E/I weights.

As expected, due to the Poisson input and the absence of any recurrent interactions, the networks have a broad ISI distribution, a relatively narrow distribution of the CV of the ISI and a Fano Factor very close to 1 (Fig Ba-c). The activity of the network is highly correlated within groups, but different groups fire independently (Fig Bd).

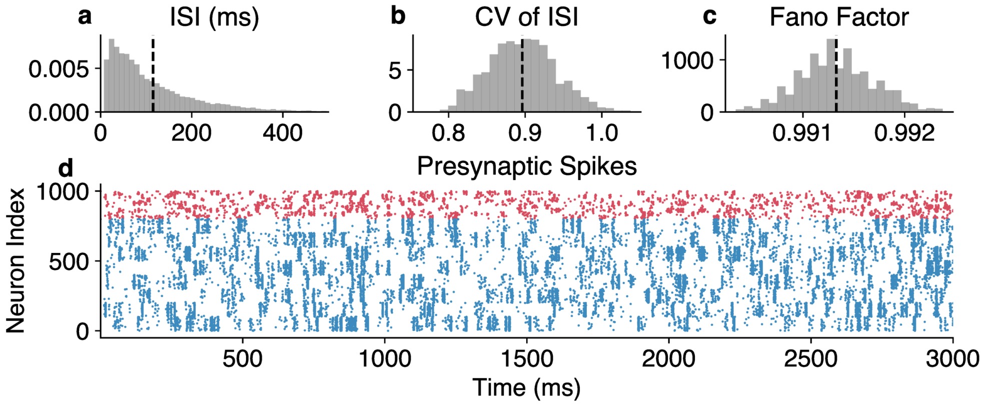

FIG. B. **The firing of a feedforward network: a.** The inter-spike interval distribution. **b.** The distribution of the Coefficient of variation ((CVs) of the ISIs (**c**)The distribution of the Fano Factor is very close to 1, indicating the Poisson input the neurons receive. (**d**) A raster plot visualizing 3 seconds of the network's activity (Here blue indicates excitatory and red inhibitory neurons)

We then examine a case that is particularly detrimental to the emergence of co-tuning. We introduce unstructured (no assemblies) recurrent connectivity ($p = 0.5$ and $W = 2$) and high noise (noise intensity is set to 0.6).

The resulting distribution of the inter-spike intervals becomes somewhat skewed, as does the distribution of the CV of the ISI, while the Fano Factor remains very close to 1 (Fig Ca-c). The network develops occasional large bursts of synchronized activity involving neurons from all groups (Fig Cd).

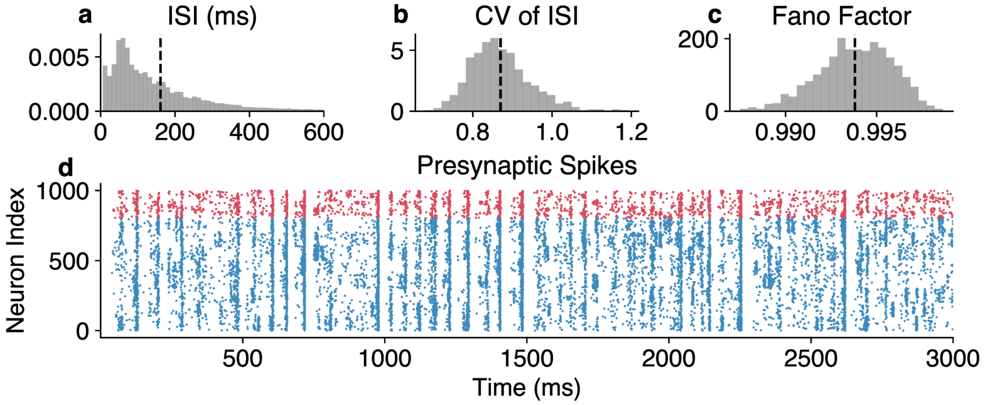

FIG. C. **The firing of a network with strong noise (noise intensity = 0.6) and strong random recurrence** ($p = 0.5$ **and** $W = 2$)**: a.** The inter-spike interval distribution. **b.** The distribution of the Coefficient of variation ((CVs) of the ISIs (**c**)The distribution of the Fano Factor remains very close to 1. (**d**) A raster plot visualizing 3 seconds of the network's activity (Here blue indicates excitatory and red inhibitory neurons)

Finally, we look at the previous network, albeit with the introduction of optimal assembly structure ($r_{EE} = r_{EI} = 1.0$ and $r_{IE} = r_{II} = 0.4$).

This network is characterized by short synchronized firing periods of each group, during which neurons of other groups are largely silent (Fig Dd). This leads to a bimodal distribution of the mean ISI and a very skewed distribution of the CV of the ISI, which indicates relatively irregular firing (largely due to the combination of bursts and longer periods of silence). Finally, the Fano Factor remains close to 1 (Fig Da-c).

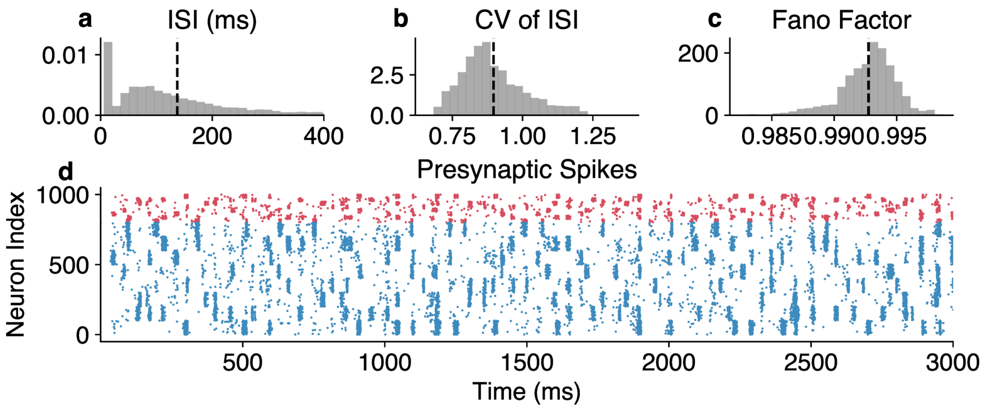

FIG. D. **The firing of a network with near-optimal clustering ($r_{EE} = r_{EI} = 1.0$ and $r_{IE} = r_{II} = 0.4$): a.** The inter-spike interval distribution. **b.** The distribution of the Coefficient of variation ((CVs) of the ISIs. The average increases slightly compared to the previous networks. (**c**)The distribution of the Fano Factor remains very close to 1. (**d**) A raster plot visualizing 3 seconds of the network's activity (Here blue indicates excitatory and red inhibitory neurons)

D. Learned connectivity and the dynamics of the post-synaptic neuron

Since synaptic plasticity depends not only on presynaptic network statistics but also on postsynaptic neuron activity, we examine the learned connectivity of different networks (the ones we examined in the last section) and visualize the activity of the postsynaptic neuron.

At first, the feedforward, low-noise network leads to a very strongly co-tuned E/I connectivity (Fig Ea) and diverse weights between different groups. The incoming currents are highly correlated (Fig Eb) and the post-synaptic neuron fires (Fig Ec) relatively sparsely ($\langle \text{ISI} \rangle = 0.398$, $\text{CV}_{\text{ISI}} = 1.15$, Fano Factor $= 0.991$).

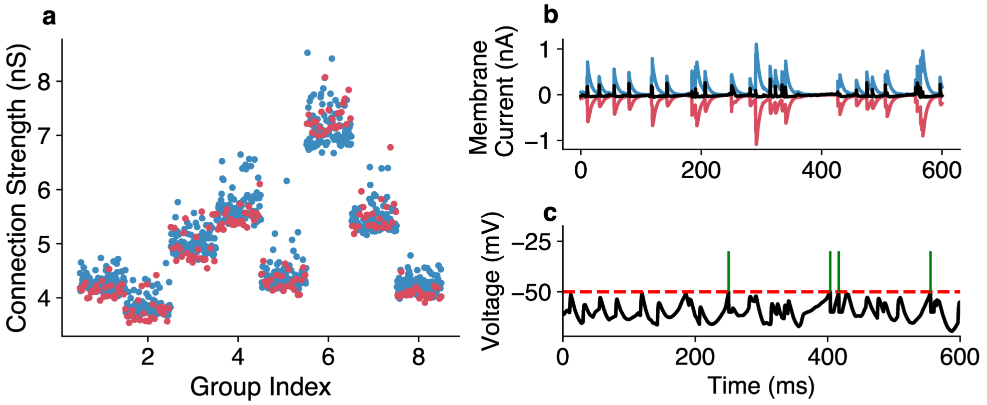

FIG. E. **Connectivity and firing of a postsynaptic neuron receiving input from a feedforward network: a.** The learned connectivity is co-tuned and diverse. **b.** The incoming currents are tightly balanced. **c** The voltage trace and spikes of the readout neuron.

On the contrary, the noisy and recurrent network fails to develop any structure in the learned connectivity. The coordinated firing across groups, visualized in (Fig Fa), leads to occasional large current influxes (Fig Fb), but otherwise, the neuron remains relatively tightly balanced. The neuron's firing (Fig Fc) becomes slightly more frequent ($\langle \text{ISI} \rangle = 0.21$, $\text{CV}_{\text{ISI}} = 1.23$, Fano Factor $= 0.991$).

Finally, the network with the optimized assembly structure, due to the restored statistics of the input (Fig Ga), develops co-tuned E/I connectivity and relatively diverse weights between groups. The currents arriving to the postynaptic neurons are tightly balanced (Fig Gb) and the firing of the postynaptic neuron (Fig Gc) becomes again more sparse and relatively irregular ($\langle \text{ISI} \rangle = 0.61$, $\text{CV}_{\text{ISI}} = 1.14$, Fano Factor $= 0.993$).

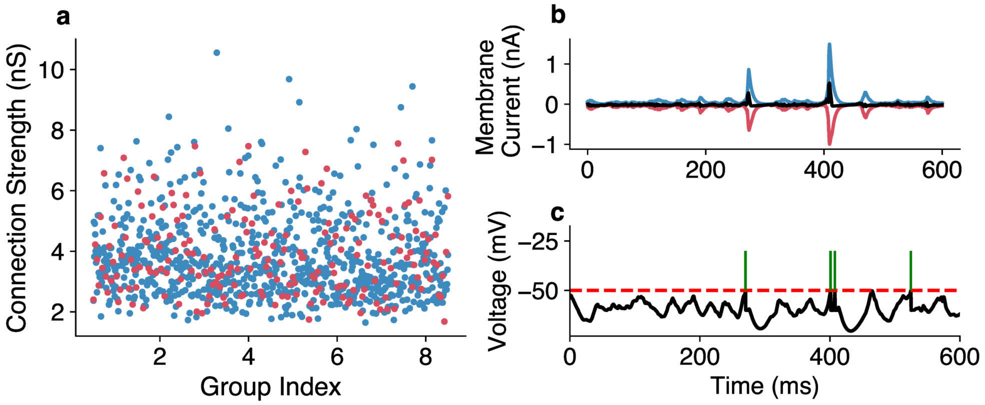

FIG. F. **Connectivity and firing of a postsynaptic neuron receiving input from a noisy, recurrent network: a.** The learned connectivity is completely unstructured. **b.** The incoming currents are tightly balanced despite occasional large influxes. **c** The voltage trace and spikes of the readout neuron.

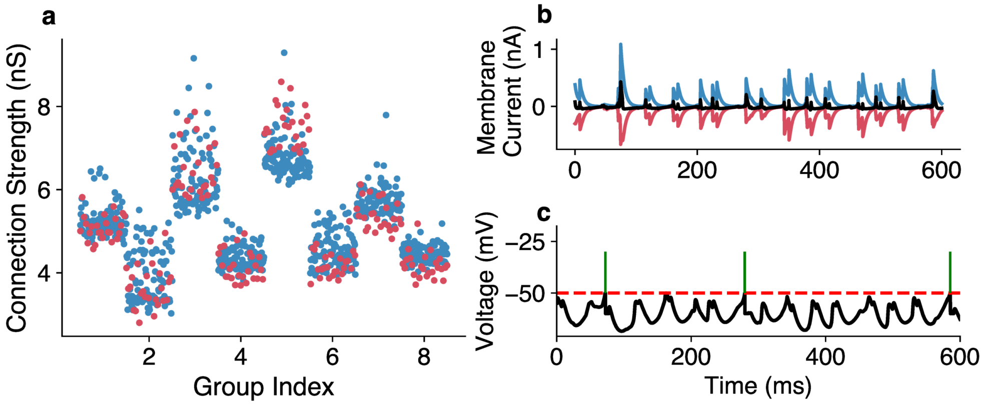

FIG. G. **Connectivity and firing of a postsynaptic neuron receiving input from a network with optimal assembly structure: a.** The learned connectivity is restored to be co-tuned and diverse. **b.** The incoming currents are tightly balanced. **c** The voltage trace and spikes of the readout neuron.

E. The effects of inhomogeneous connectivity are independent of the plasticity protocols' details

We examined whether our results are dependent on the particular plasticity protocol we used, and we verified that they hold for alternative plasticity mechanisms. Specifically, we tested whether a variety of different plasticity protocols produces co-tuning in the simple feedforward case, whether the effects of noise and recurrence on the resulting connectivity are consistent, and whether the inferred optimal fixed pre-synaptic recurrent connectivity restores the ability of the plasticity to produce co-tuning.

Starting with the plasticity protocol we used in our original experiments, we visualize the development of excitatory (Fig Ha) and inhibitory weights (Fig Hb), the matching resulting connectivity (Fig Hc) and the incoming E/I currents to the post-synaptic neuron after convergence (Fig Hd) in a setting with optimal connectivity for comparison with the other plasticity protocols simulated with the same connectivity and noise levels.

i. Excitatory plasticity: Parameters of the Triplet rule

In our experiments presented in the main text, we have used a simplified form of the triplet STDP rule for the excitatory synapses, which relies on identical slow and fast timescales for the pre and postsynaptic traces. We first tested how robust our results are to changes on the LTD/LTP ratio (varying the $A_{LTD} \in [0.05, 1.2]$ and $A_{LTP} \in [0.05, 1.0]$) as well as the timescale of the fast and slow traces. We found that qualitatively our results are robust to these changes in the plasticity protocol.

Moreover, in order to ensure that our results did not depend on the particular form of the rule we used, we

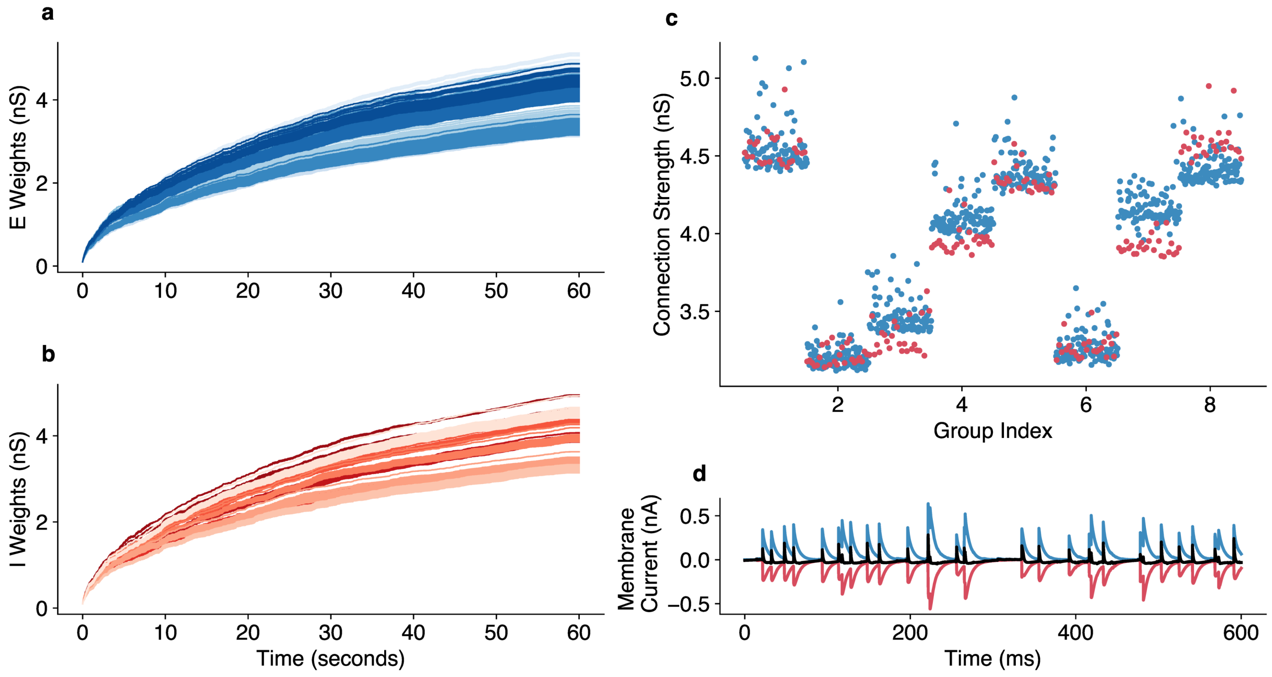

FIG. H. **Weight development under the original protocol.** The development of **a.** excitatory and **b.** inhibitory weights over a minute of simulation time. **c.** The convergence point of excitatory (blue) and inhibitory weights (red). The inhibitory weights are scaled to account for the slower synaptic constant of the inhibitory synapses and the smaller number of inhibitory neurons. **d.** The resulting E/I currents on the postsynaptic neuron are tightly balanced.

replicated our main simulations using the original implementation of the triplet rule from [3], which implemented different timescales for the pre and postsynaptic fast and slow traces.

Specifically, in this implementation, the pre and post-synaptic activity is tracked by the traces:

$$\frac{dy_k^E(t)}{dt} = \frac{-y_k^E(t)}{\tau_{\text{pre}}^{\text{fast}}} + \sum_f \delta(t - t_k^f), \tag{2a}$$

$$\frac{dz_k^E(t)}{dt} = \frac{-z_k^E(t)}{\tau_{\text{pre}}^{\text{slow}}} + \sum_f \delta(t - t_k^f), \tag{2b}$$

$$\frac{dx_1(t)}{dt} = \frac{-x_1(t)}{\tau_{\text{post}}^{\text{fast}}} + \sum_f \delta(t - t_x^f), \tag{2c}$$

$$\frac{dx_2(t)}{dt} = \frac{-x_2(t)}{\tau_{\text{post}}^{\text{slow}}} + \sum_f \delta(t - t_x^f), \tag{2d}$$

where $\tau_{\text{pre}}^{\text{fast}} = 16.8$ ms, $\tau_{\text{pre}}^{\text{slow}} = 101$ ms, $\tau_{\text{post}}^{\text{fast}} = 33.7$ ms and $\tau_{\text{post}}^{\text{slow}} = 125$ ms following the parameters from [3, 4]. Additionally, $y_k^E(t)$, $z_k^E(t)$ and $x_1(t)$, $x_2(t)$ represent the slow and fast traces of the k-th excitatory pre-synaptic and the single post-synaptic neuron respectively while t_k^f and t_x^f are their respective firing times. The function $\delta(x)$ represents a Dirac's delta. The connection weights are updated upon pre and post-synaptic spiking according to

$$\Delta W_k^E = \eta_E \cdot y_k^E(t) \cdot (A_2^+ + A_3^+ \cdot x_2(t)) \cdot \sum_f R(t - t_x^f) - \eta_E \cdot x_1(t) \cdot (A_2^- + A_3^- \cdot z_k^E(t)) \sum_f R(t - t_k^f), \tag{3}$$

where $A_2^- = 0.7$, $A_3^- = 0.023$, $A_2^+ = 7.5 \cdot 10^{-8}$, $A_3^+ = 0.93$ and $\eta_E = 10^{-2}$, following the parameters of [3, 4]. We see that under this protocol (Fig Ia, b), the weights develop very similarly to the one we used for our experiments (Fig Ha, b) under optimal connectivity. Moreover, we see that strong co-tuning emerges (Fig Ic) as well as tightly balanced E/I currents to the postsynaptic neuron as expected (Fig Id).

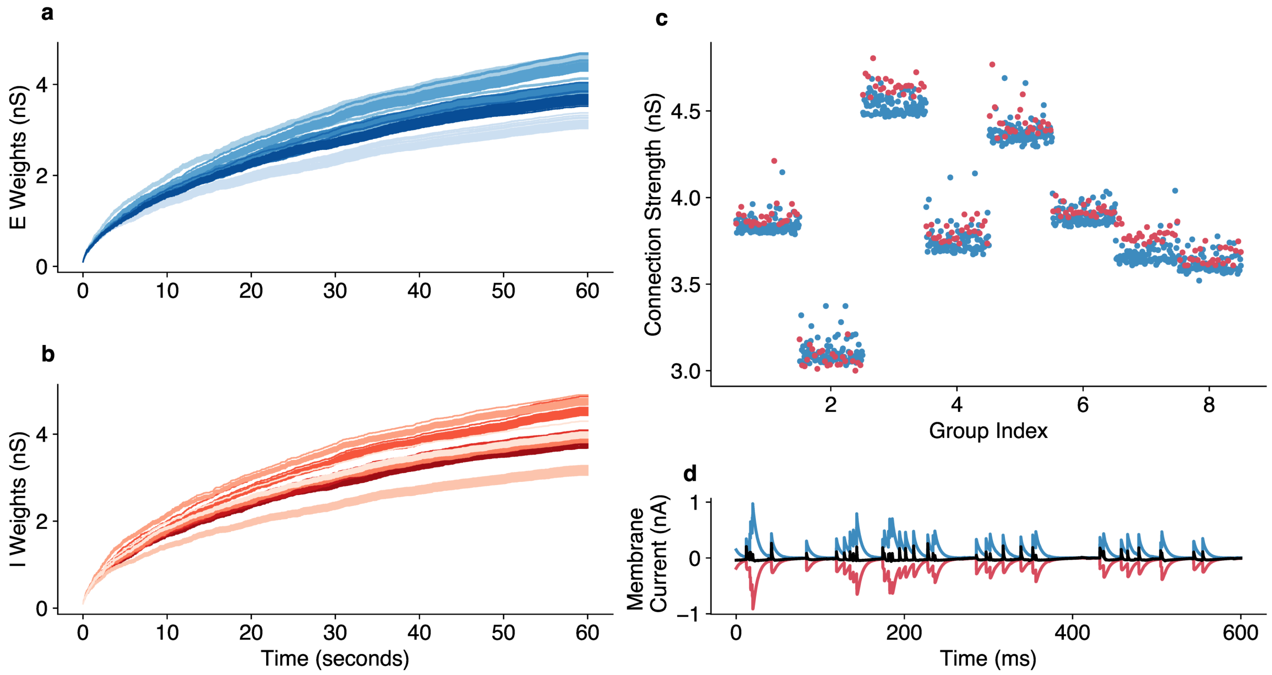

FIG. I. **Weight development with the original triplet rule parameters.** The development of **a.** excitatory and **b.** inhibitory weights over a minute of simulation time. **c.** The convergence point of excitatory (blue) and inhibitory weights (red). The inhibitory weights are scaled to account for the slower synaptic constant of the inhibitory synapses and the smaller number of inhibitory neurons. **d.** The resulting E/I currents on the postsynaptic neuron are tightly balanced.

ii. Excitatory plasticity: Triplet vs Pair rule

We further examined whether a different Hebbian learning rule in the excitatory synapses has any impact on our results. In particular, we replaced the triplet rule [3] with a classic spike pair Hebbian rule [5], which relies on a single pre and postsynaptic trace:

$$\frac{dy_k^E(t)}{dt} = \frac{-y_k^E(t)}{\tau} + \sum_f \delta(t - t_k^f), \tag{4a}$$

$$\frac{dx(t)}{dt} = \frac{-x(t)}{\tau} + \sum_f \delta(t - t_x^f), \tag{4b}$$

where $y_k^E(t)$ the trace of the k-th presynaptic excitatory neuron, $x(t)$ the trace of the postsynaptic neuron and $\tau = 10$ ms the time constant of the decay. The weight update happens as:

$$\Delta W_k^E = \eta_E \cdot A_{LTP} \cdot y_k^E(t) \cdot \sum_f R(t - t_x^f) - \eta_E \cdot A_{LTD} \cdot x(t) \sum_f R(t - t_k^f), \tag{5}$$

where $A_{LTP} = 1.0$ and $A_{LTD} = 0.2$ same as for the simplified triplet rule. Using the same competitive, synapse-type specific normalization, we found that in this setting, the weight development as well as the impact of noise and recurrence are similar to our original setting.

iii. Inhibitory learning rule target rate

We additionally examined several different target rates ρ_0 (ranging from 0 to 6 Hz) for the inhibitory plasticity rule (setting the rate to $\rho_0 = 0$, i.e., making the inhibitory plasticity into a pure symmetric Hebbian rule, as was used in

the original study of the normalization mechanism by [6]). Besides an expected change in the postsynaptic firing rate after the convergence of the weights, we did not observe any other noticeable changes in our findings regarding the emergence of co-tuning and input selectivity.

iv. Alternative implementations of the competitive Normalization

1. Regular vs event-based normalization steps

For reasons of numerical simulation speed, we implemented the weight normalisation in an asynchronous manner. Specifically, we apply a normalisation step after every weight update occurs (i.e., after each postsynaptic spike all connections get normalised and after each presynaptic spike the corresponding connection gets normalised). Since the presynaptic population has a homogeneous firing rate (i.e., all neurons spike approximately the same number of times) all connections are normalised approximately equally often, which makes this implementation behave similarly to implementing the normalisation step on each time step of the simulation.

To test that this assumption is correct, we simulated a network with a regular normalization update, where all connections are normalized simultaneously every time step ($dt = 0.1$ ms). Besides a modification of the rate of the normalization η_N (necessary to counter the higher frequency of normalization in the regular case), we maintain the exact same parameters as in the original experiment.

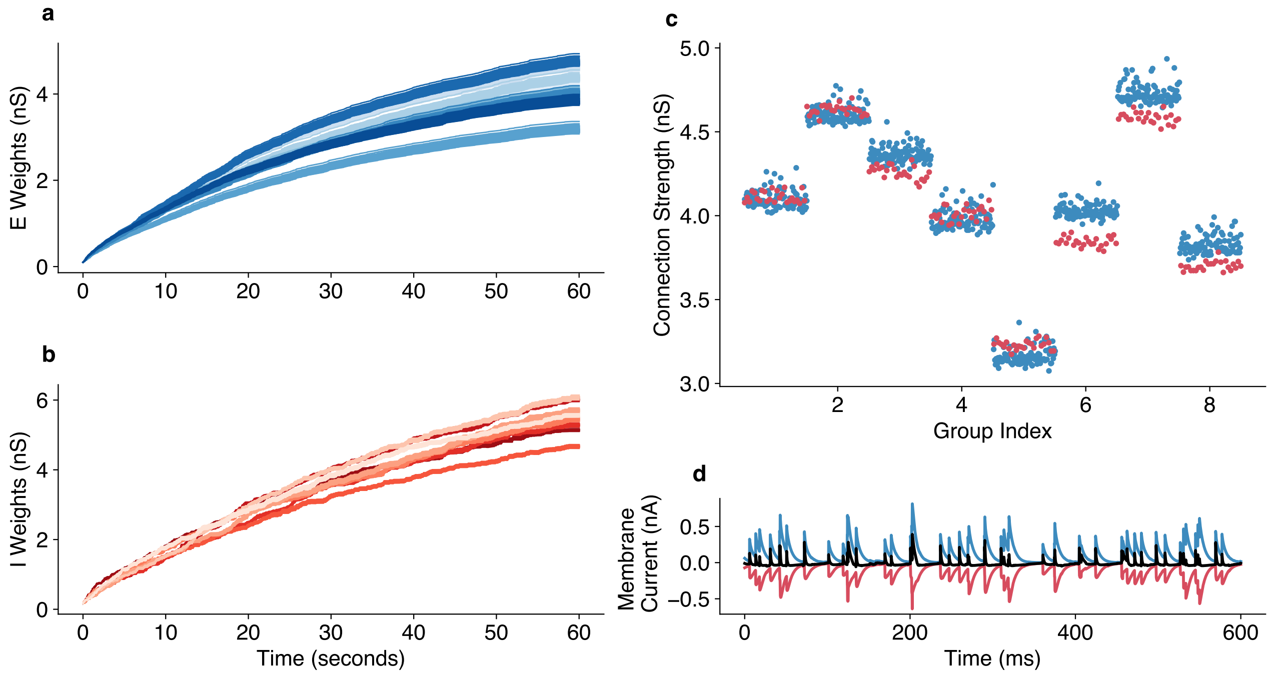

FIG. J. **Weight development with a regular normalization update.** The development of **a.** excitatory and **b.** inhibitory weights over a minute of simulation time. **c.** The convergence point of excitatory (blue) and inhibitory weights (red). The inhibitory weights are scaled to account for the slower synaptic constant of the inhibitory synapses and the smaller number of inhibitory neurons. **d.** The resulting E/I currents on the postsynaptic neuron are tightly balanced.

We observe that in this setting, the weight development happens very similar to the original experiment (Fig Ja, b), producing co-tuning in cases with optimal input connectivity (Fig Jc) and tight E/I current balance (Fig Jd). In summary, we find that the two normalization methods behave similarly for our setting, suggesting that our findings are independent of how the weight normalization is implemented. However, in settings different from ours, where the pre-synaptic rates are largely inhomogeneous, we would expect that the two normalization methods would produce different results (since some weights would be normalized more often than others), which might lead to different weight dynamics. Thus, the numerical convenience of the asynchronous normalization updates cannot be generally used in simulations of other types of networks with potentially very different distributions of firing rates.

2. "Soft" vs. "Strict" normalization: The impact of the normalization rate

As described in the "Methods" section of the main text, we implement our normalization step as follows:

$$W_k^A(t) \leftarrow (1-\eta_N)\cdot W_k^A(t) + \eta_N \cdot W_k^A(t)\cdot \frac{W_{target}^A}{\sum_{i=1}^{N_A} W_i^A(t)},\ A \in \{E, I\}. \tag{6}$$

where $\eta_N = 3\cdot 10^{-3}$ to match the equivalent excitatory and inhibitory learning rates. This essentially implements a "soft" normalization, where the total sum of the incoming weights to the postsynaptic neuron is not kept constant in each timestep but rather pushed towards maintaining a sum as close as possible to the target weight sum over time.

However, if we treated the normalization strictly as the preservation of synaptic resources over time following [6], we would need to set $\eta_N = 1$, in order to preserve the exact weight sum constant for each time step. This approach would impose a very strict condition, which, in combination with the small learning rates of the E and I plasticity, would prevent weight diversification.

One way of countering this is by massively increasing the E and I learning rates, but this tends to make the learning dynamics unstable. Another way of solving this problem is by implementing the normalisation regularly but not on every time step (a solution that has been used previously in [4]), for example, implementing it every 20 ms. This does lead to stable learning, but it also occasionally promotes winner-take-all connectivity, which is not in itself problematic, but may be undesirable for some types of coding. The emergence of winner-take-all connectivity can, in turn, be potentially countered by faster inhibitory plasticity relative to the excitatory plasticity, but finding the exact parameters can involve a fair amount of fine-tuning. For our experiment, the "soft" which can be biologically justified as a competition for synaptic resources among multiple neurons, happening on a very slow timescale, clearly leads to more plausible and stable results.

v. Subtractive Normalization with modified input

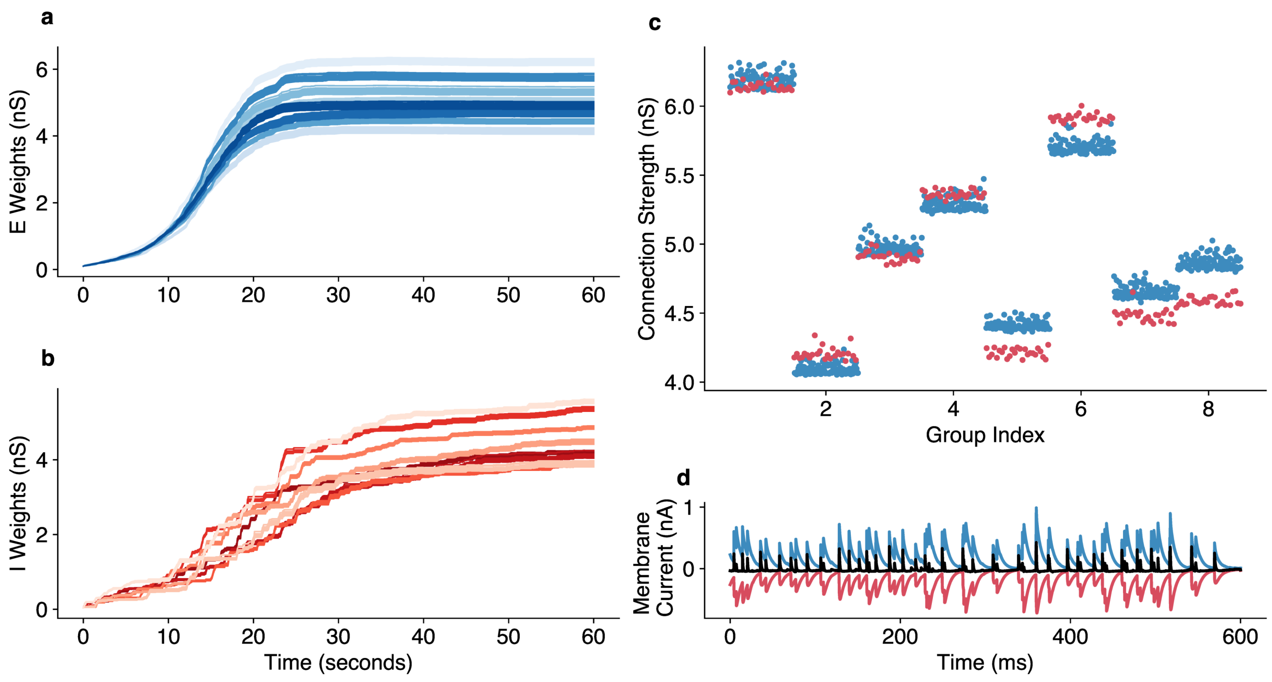

FIG. K. **Alternative normalization protocol.** The development of **a.** excitatory and **b.** inhibitory weights over a minute of simulation time. **c.** The convergence point of excitatory (blue) and inhibitory weights (red). The inhibitory weights are scaled to account for the slower synaptic constant of the inhibitory synapses and the smaller number of inhibitory neurons. **d.** The resulting E/I currents on the postsynaptic neuron are tightly balanced.

Finally, in order to fully demonstrate that our results are independent of the exact normalization protocol, we studied both the triplet [3] and pair [5] excitatory Hebbian rules combined with a subtractive normalization mechanism that

has been previously used in plasticity studies [4, 7]. Following our setting for the multiplicative normalization, we applied this rule also as a “soft” normalization in the excitatory synapses (the inhibitory synapses are not normalized in this setting):

$$W_k^E(t) \leftarrow (1-\eta_N)\cdot W_k^E(t) + \eta_N \cdot W_k^E(t)\cdot \frac{\sum_{i=1}^{N_E} W_i^E - W^{\text{target}}}{N_E}. \tag{7}$$

In 2016 [8], it was demonstrated that subtractive normalization only on the excitatory connections will lead to all the weights converging on the same point due to the inhibitory plasticity creating a moving threshold. In order to prevent this collapse of the receptive field, enforced inhomogeneity on the firing rates of different groups is needed. We solved this problem by giving the network's input as pulses of 100 mS during which some of the input groups firing rate quadruples. This enforces inhomogeneous firing rates, which result in the emergence of stable, diverse, and co-tuned feedforward connectivity (Fig Ka - c). We also verified that the post-synaptic neuron is tightly balanced (Fig Kd) and maintains a stable firing rate.

We verified that the co-tuning achieved in this setting, similar to the mechanism presented in the main text, suffers from the introduction of noise and recurrent connectivity. Furthermore, the assembling principles that we derived for the original network seem to have a similarly beneficial effect on this setting, restoring the original covariance structure of the network's activity and leading to detailed co-tuning between the excitatory and inhibitory feedforward connections.

F. Convergence of weights to an eigenvector of a modified covariance matrix under plasticity

The study that introduced the competitive synapse type-specific normalization [6] analytically predicted that for Hebbian E and I feedforward plasticity, the convergence point of the weights is an eigenvector of the modified covariance matrix (Fig La):

$$\overline{C} = \left\langle \begin{pmatrix} EE^T & -EI^T \\ IE^T & -II^T \end{pmatrix} \right\rangle \tag{8}$$

where E, I are the activities of the excitatory and inhibitory populations, respectively.

We test whether in our modified plasticity protocol (simplified Triplet STDP in E connections, slower normalization), the convergence point can be approximated by an eigenvector of the above matrix.

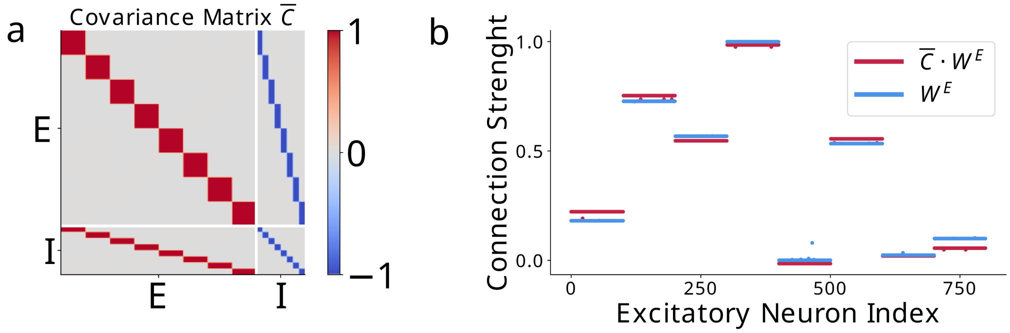

FIG. L. **Weights converge to an eigenvector of the covariance matrix.** **a**: The estimated covariance matrix for a feedforward network. **b**. We verify that the convergence point of the weights is an eigenvector of the covariance matrix.

Specifically, we multiply the converged weight vector with a numerical calculation of the covariance (estimated via binning of the spike trains with a bin size of 1 mS), for different noise and recurrence settings, and we verify that the resulting product is approximately equal to a multiple of the original weight matrix (Fig Lb). This indicates that despite the differences in the learning protocol, the plasticity converge point is largely controlled by the covariance structure of the population activity.

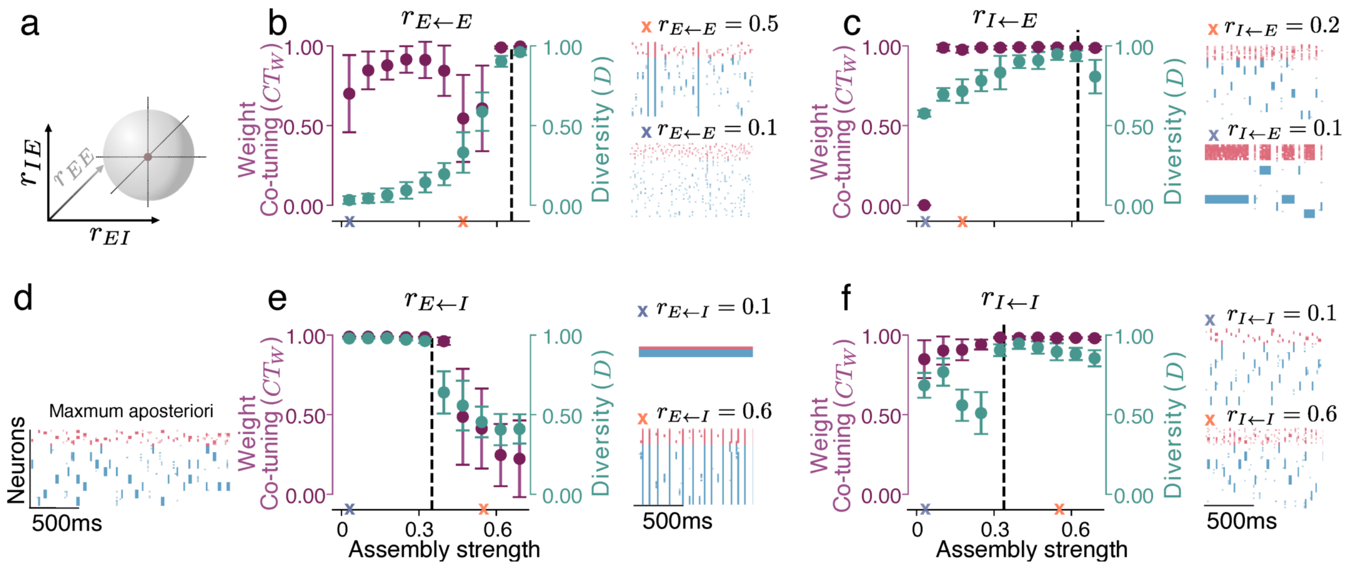

FIG. M. **Changes of the various assemblies strengths differently affect weight co-tuning and diversity. a.** We sequentially vary the value of each assembly strength while keeping the rest of the parameters fixed at the maximum a posteriori (MAP) solution (**d**). **b.** A decrease in the $E \leftarrow E$, assembly strength ($R_{E\leftarrow E}$) introduces synchronous burst-like events that jeopardize co-tuning and weight diversity. Further reduction of the $E \leftarrow E$ assembly strength results in sparse, asynchronous spiking, which significantly reduces tuning quality. **c.** Reduction of $I \leftarrow E$ assembly strength first leads to synchronous inhibitory firing across groups, and further reduction leads to persistent activity of the whole inhibitory population combined with bursts of excitatory activity that prevent the development of diversity. **e.** Decreasing the $E \leftarrow I$ assembly strength leads to persistent activation of a single group of neurons (which affects the tuning only marginally). While the increase of the $E \leftarrow I$ assembly strength leads to synchronized behavior of the whole network. **f.** Weakening the $I \leftarrow I$ assemblies decreases the weight diversity by introducing occasional synchronous bursts in the network while strengthening them leads to asynchronous inhibitory activity.

G. Perturbation of the optimal assembly strengths leads to diverse effects on the network's activity

To investigate the relative importance of the different connection types, we perturb various assemblies away from the optimal solutions inferred with ABC and check the resulting changes on the network activity as well as how fast the weight co-tuning and diversity deteriorate.

We find that $E \leftarrow I$ assemblies have a strong impact on the resulting network dynamics and, consequently, the statistics that determine the emergence of co-tuning (Fig Me). Thus, when $E \leftarrow I$ assemblies are too strong, the network exhibits synchronous behaviour that does not allow discrimination between neuron groups and, consequently, the emergence of co-tuned connectivity. In contrast, when $E \leftarrow I$ assemblies are too weak, one group of neurons is constantly active. This eventually leads to perfect discrimination of only a single input.

$E \leftarrow E$ assemblies also have a strong effect on the resulting learned connectivity. When they are reduced, the weight diversity rapidly reduces, while the weight co-tuning metric first decreases and then restores. This behaviour is related to two different bifurcations in the population dynamics. First, synchronous full-network bursts emerge on top of the stimulus-induced firing (Fig Ma). Then, as $E \leftarrow E$ are reduced further, the network enters an asynchronous irregular state (Fig Ma).

Perturbations of $I \leftarrow I$ and $I \leftarrow E$ assemblies have a weaker effect on the weight co-tuning and diversity. Specifically, when $I \leftarrow I$ assemblies are weakened, the network displays synchronous bursts on top of the stimulus-driven activity that only minimally reduces the weight co-tuning and diversity (Fig Md). When $I \leftarrow E$ assemblies are reduced, the weight diversity slowly decreases due to a decrease in the diversity of inhibitory weights. Thus, the activity of the inhibitory population becomes more discoordinated (Fig Mc). On the other hand, a network with uniform $I \leftarrow E$ connections (i.e., without $I \leftarrow E$ assemblies), shows a dramatic reduction in the weight co-tuning metric because of very strong activation of a single neuron group accompanied by the simultaneous activity of all inhibitory neurons (Fig Mc).

H. Reduced model calculations

i. Derivation of the equations

In this Appendix, we start from the main text Eqs. 14,

$$\dot{x}_i = a x_i + b y_i + \frac{1}{M-1}\sum_{j\neq i}\left(W^{E\leftarrow E}x_j + W^{E\leftarrow I}y_j\right) + \sigma_{\text{int}}\xi_i^x(t) + \sigma_{\text{ext}}\eta_i(t) + \eta_0, \tag{9a}$$

$$\dot{y}_i = c x_i + d y_i + \frac{1}{M-1}\sum_{j\neq i}\left(W^{I\leftarrow E}x_j + W^{I\leftarrow I}y_j\right) + \sigma_{\text{int}}\xi_i^y(t) + \sigma_{\text{ext}}\eta_i(t) + \eta_0. \tag{9b}$$

where we consider M groups composed by excitatory $x_i(t)$ and inhibitory $y_i(t)$ populations ($i = 1, \ldots, n$) coupled linearly (see also Methods). Internal noise of each population is represented by $\xi_i^x(t)$ and $\xi_i^y(t)$ for excitatory and inhibitory populations, respectively. On the other hand, external noise $\eta_i(t)$ is shared among both populations. All the noises have zero mean and correlations

$$\left\langle \xi_i^c \xi_j^{c'} \right\rangle = \delta_{cc'}\delta_{ij}\delta(t-t'), \tag{10a}$$

$$\left\langle \xi_i^c(t)\,\eta_j(t') \right\rangle = 0 \quad \forall i, j, t, t', \tag{10b}$$

$$\left\langle \eta_i(t)\,\eta_j(t') \right\rangle = \delta_{ij}\delta(t-t'). \tag{10c}$$

We will derive closed expressions for the correlation coefficients. We would like to remark that $\langle\cdot\rangle$ means an ensemble average over noise realizations. All stochastic equations are to be interpreted in the Itô convention[9].

First of all, we redefine the noise terms, which will prove convenient later to simplify the algebra. For this reason, we define

$$\xi_i^1(t) = \sigma_{\text{int}}\xi_i^x(t) + \sigma_{\text{ext}}\eta_i(t), \tag{11a}$$

$$\xi_i^2(t) = \sigma_{\text{int}}\xi_i^y(t) + \sigma_{\text{ext}}\eta_i(t), \tag{11b}$$

which are Gaussian white noises with zero mean and correlation matrix

$$\left\langle \xi_i^1(t)\,\xi_j^1(t') \right\rangle = \left\langle \xi_i^2(t)\,\xi_j^2(t') \right\rangle = \delta_{ij}\delta(t-t')\left(\sigma_{int}^2 + \sigma_{ext}^2\right), \tag{12a}$$

$$\left\langle \xi_i^1(t)\,\xi_j^2(t') \right\rangle = \delta_{ij}\delta(t-t')\,\sigma_{ext}^2. \tag{12b}$$

To start with, one can obtain the average values for the stationary rates by applying averages to both sides of equations (9) and imposing the stationary state condition, $\langle \dot{x}_i\rangle = \langle \dot{y}_i\rangle = 0$. Once this is done, it is immediate to solve the resulting linear system and check that $\langle x_i^*\rangle, \langle y_i^*\rangle \propto \eta_0$, where the star (*) indicates that these values correspond to the stationary state. Hence, making $\eta_0 = 0$ the mean values vanish. One can demonstrate that correlations do not depend on η_0, and hence we can make $\eta_0 = 0$ without loss of generality. Conceptually, this means just shifting up or down the baseline of fluctuations of the firing rate, which does not affect the fluctuations themselves.

To compute correlations, we need to evaluate the second-order moments between different populations as $\langle x_i y_j\rangle$ or $\langle x_i^2\rangle$. A possible way of doing this is starting from the analytical solution of the multidimensional Orstein-Uhlenbeck process [10]. However, this approach will yield a linear system with $N(N+1)/2$ variables to solve for, which are all the elements of the (symmetric) correlation matrix. But all the groups are identical (or *indistinguishable*), so we expect correlations not to depend on the particular population chosen. Therefore, all the equations will be reduced to just 6 covariances: $\langle x_i x_j\rangle$, $\langle x_i y_j\rangle$, $\langle y_i y_j\rangle$, $\langle x_i^2\rangle$, $\langle x_i y_i\rangle$ and $\langle y_i^2\rangle$.

In this context, it is conceptually simpler to obtain equations for the evolution of the second moments and then evaluate them in the stationary state. Here, we report in detail the computation of two of these moments as an example, giving just the final answer for the other four, which is performed in an analogous way.

First, we define $X_{ij} = x_i x_j$, and then look for the time evolution of X_{ij}, i.e., $\dot{X}_{ij}$. Notice that this is a non-linear change of variables, and thus Itô's lemma is required. The lemma tells us that if we have a change of variables $z = z(x)$, then one has to include the second-order terms in the expansion,

$$dz = \underbrace{\sum_{i=1}^{N}\partial_{x_i} z\, dx_i}_{\text{Chain rule}} + \underbrace{\frac{1}{2}\sum_{i=1}^{N}\partial_{x_i}\partial_{x_j} z\, dx_i dx_j}_{\text{Itô's lemma}}. \tag{13}$$

The terms dx_i can be obtained as $\dot{x}_i dt$. It is important to remark that in this procedure noise terms are rewritten as the differential of the Wiener processes, i.e., $\eta_i(t)dt = dW_i$. After applying the Itô lemma above, only terms up to order dt should be taken into account. Notice that $dW_i(t) \propto \sqrt{dt}$ [10]. Finally, one just divides again by dt to recover the stochastic differential equation and applies the ensemble average.

For X_{ij}, this reads as

$$\frac{d\left\langle x_i x_j\right\rangle}{dt} = \left\langle \dot{x}_i x_j\right\rangle + \left\langle x_i \dot{x}_j\right\rangle + \frac{1}{2}\left\langle \dot{x}_i \dot{x}_j\right\rangle = \\ a\left\langle x_i x_j\right\rangle + b\left\langle y_i x_j\right\rangle + \frac{W^{E\leftarrow E}}{M-1}\sum_{k\neq i}\left\langle x_k x_j\right\rangle + \frac{W^{E\leftarrow I}}{M-1}\sum_{k\neq i}\left\langle y_k x_j\right\rangle + \\ + a\left\langle x_i x_j\right\rangle + b\left\langle x_i y_j\right\rangle + \frac{W^{E\leftarrow E}}{M-1}\sum_{k\neq j}\left\langle x_i x_k\right\rangle + \frac{W^{E\leftarrow I}}{M-1}\sum_{k\neq j}\left\langle x_i y_k\right\rangle + \\ \left\langle \xi_i^1 x_j\right\rangle + \left\langle x_i \xi_j^1\right\rangle + \left\langle \xi_i^1 \xi_j^1\right\rangle + \mathcal{O}\left(dt^2\right), \quad (14)$$

where all the averages between the noise and the variable yield 0, due to Itô's prescription. The next step is to simplify the sums involving correlations. As discussed above, since clusters are indistinguishable, all the terms are exactly the same. However, the dummy index k will also take the value of the fixed index, $k = j$, and this has to be taken into account separately since the in-group is different to the between-group one. Then,

$$\sum_{k\neq i}\left\langle x_k x_j\right\rangle = (M-2)\left\langle x_i x_j\right\rangle + \left\langle x_i^2\right\rangle, \quad (15)$$

allowing us to simplify the equation. At this step we simplify the notation by letting $X_{ij} = \langle x_i x_j\rangle$, $Z_{ij} = \langle x_i y_j\rangle$, $Y_i = \langle y_i^2\rangle$, etc., leading to

$$\frac{1}{2}\dot{X}_{ij} = \left(a + \frac{M-2}{M-1}W^{E\leftarrow E}\right)X_{ij} + \left(b + \frac{M-2}{M-1}W^{E\leftarrow I}\right)Z_{ij} + \frac{1}{M-1}\left[W^{E\leftarrow E}X_i + W^{E\leftarrow I}Z_i\right]. \quad (16)$$

The same procedure can be repeated for all the other correlations, such as

$$\frac{d\left\langle y_i^2\right\rangle}{dt} = \left\langle 2y_i\dot{y}_i\right\rangle + \frac{1}{2}\left\langle 2\dot{y}_i^2\right\rangle = \\ 2c\left\langle x_i y_i\right\rangle + 2d\left\langle y_i^2\right\rangle + \frac{2W^{I\leftarrow E}}{M-1}\sum_{k\neq i}\left\langle y_i x_k\right\rangle + \frac{2W^{I\leftarrow I}}{M-1}\sum_{k\neq i}\left\langle y_i y_k\right\rangle + 2\left\langle y_i \xi_i^2\right\rangle + \left\langle \xi_i^2 \xi_i^2\right\rangle = \\ 2c\left\langle Z_i\right\rangle + 2d\left\langle Y_i\right\rangle + 2W^{I\leftarrow E}\left\langle Z_{ij}\right\rangle + 2W^{I\leftarrow I}\left\langle Y_{ij}\right\rangle + \sigma_{\text{int}}^2 + \sigma_{\text{ext}}^2, \quad (17)$$

where now the correlation between noises yields a non-vanishing value. This operation is repeated with all the remaining terms, in order to find a linear system of differential equations with 6 variables and 6 equations,

$$\frac{1}{2}\dot{X}_i = aX_i + bZ_i + \left[W^{E\leftarrow E}X_{ij} + W^{E\leftarrow I}Z_{ij}\right] + \frac{1}{2}\left(\sigma_{\text{int}}^2 + \sigma_{\text{ext}}^2\right), \quad \text{(18a)}$$

$$\frac{1}{2}\dot{Y}_i = cZ_i + dY_i + \left[W^{I\leftarrow E}Z_{ij} + W^{I\leftarrow I}Y_{ij}\right] + \frac{1}{2}\left(\sigma_{\text{int}}^2 + \sigma_{\text{ext}}^2\right), \quad \text{(18b)}$$

$$\dot{Z}_i = cX_i + (a+d)Z_i + bY_i + \left[W^{I\leftarrow E}X_{ij} + \left(W^{E\leftarrow E} + W^{I\leftarrow I}\right)Z_{ij} + W^{E\leftarrow I}Y_{ij}\right] + \sigma_{\text{ext}}^2, \quad \text{(18c)}$$

$$\frac{1}{2}\dot{X}_{ij} = \left(a + \frac{M-2}{M-1}W^{E\leftarrow E}\right)X_{ij} + \left(b + \frac{M-2}{M-1}W^{E\leftarrow I}\right)Z_{ij} + \frac{1}{M-1}\left[W^{E\leftarrow E}X_i + W^{E\leftarrow I}Z_i\right], \quad \text{(18d)}$$

$$\frac{1}{2}\dot{Y}_{ij} = \left(d + \frac{M-2}{M-1}W^{I\leftarrow I}\right)Y_{ij} + \left(c + \frac{M-2}{M-1}W^{I\leftarrow E}\right)Z_{ij} + \frac{1}{M-1}\left[W^{I\leftarrow I}Y_i + W^{I\leftarrow E}Z_i\right], \quad \text{(18e)}$$

$$\dot{Z}_{ij} = \left(c + \frac{M-2}{M-1}W^{I\leftarrow E}\right)X_{ij} + \left(b + \frac{M-2}{M-1}W^{E\leftarrow I}\right)Y_{ij} + \left(a + d + \frac{M-2}{M-1}\left(W^{E\leftarrow\leftarrow E} + W^{I\leftarrow\leftarrow I}\right)\right)Z_{ij} + \quad \text{(18f)}$$

$$+ \frac{1}{M-1}\left[W^{I\leftarrow E}X_i + W^{E\leftarrow I}Y_i + \left(W^{E\leftarrow E} + W^{I\leftarrow I}\right)Z_i\right]. \quad \text{(18g)}$$

This system can be solved in the stationary limit when all the derivatives of the left-hand side are zero. From these, one is able to obtain the Pearson correlation coefficients. Correlation with itself is always unity, thus there are only four coefficients remaining: the correlation between excitation and inhibition inside a group $C_{EI}^{\text{int}} = Z_i^* / \sqrt{X_i^* Y_i^*}$, and all three between-group correlations, $C_{EE}^{\text{ext}} = X_{ij}^*/X_i^*$, $C_{II}^{\text{ext}} = Y_{ij}^*/Y_i^*$, and $C_{EI}^{\text{ext}} = Z_{ij}^*/\sqrt{X_i^* Y_i^*}$.

ii. Solutions for the homogeneous network

In some special cases, it is possible to give a simple solution in closed form for the correlation coefficients. One example is the homogeneous network: when all weights are identical, and an intrinsic decay is added to both the excitatory and inhibitory populations (i.e., with $c = W^{E\leftarrow E} = W^{I\leftarrow E} = +W$, $b = W^{I\leftarrow E} = W^{I\leftarrow I} = -W$ and $a = W-1$, $d = -W-1$) the solution reads

$$C_{EI}^{\text{int}} = \frac{r^2 (M-1)^2 + W^2 (1-r)^2 M}{\sqrt{M^2 (1-r)^4 W^4 + (M-1)(1-r)^2 W^2 \left[M(2-4(1-r)r) - (1-r)^2\right] + (M-1)^4 \left[2(r-1)r+1\right]^2}}, \tag{19a}$$

$$C_{EI}^{\text{ext}} = \frac{W^2 (1-r)^2 M}{\sqrt{M^2 (1-r)^4 W^4 + (M-1)(1-r)^2 W^2 \left(M(4(r-1)r+2) - (r-1)^2\right) + (M-1)^4 (2(r-1)r+1)^2}}, \tag{19b}$$

$$C_{EE}^{\text{ext}} = \frac{(1-r)^2 W \left((W+1)M - 1\right)}{M(1-r)^2 W^2 + (M-1)(1-r)^2 W + (1-M)^2 \left[1 - 2(1-r)r\right]}, \tag{19c}$$

$$C_{II}^{\text{ext}} = \frac{(1-r)^2 W \left((W-1)M + 1\right)}{M(1-r)^2 W^2 - (M-1)(1-r)^2 W + (1-M)^2 \left[1 - 2(1-r)r\right]}, \tag{19d}$$

where we defined r as the signal-to-noise ratio, $\sigma_{\text{int}} = r\sigma$, $\sigma_{\text{ext}} = (1-r)\sigma$. This analytical solution has some interesting features. First, notice it does not depend on the total amount of noise σ that the system receives, but only on the ratio between external and internal noise. Second, if $W \to \infty$ all correlations go to 1, making the diversity between groups vanish. Expanding in series around $\epsilon = 1/W = 0$, one can see that all coefficients are $C = 1 - \mathcal{O}(1/W^2)$ for large coupling.

It is also possible to study the limiting values of the noise. $r = 1$ makes all the between-group correlations equal to zero, while coupling determines the in-group value. On the other hand, when $r \ll 1$, one gets

$$C_{EI}^{\text{int}} \simeq C_{EI}^{\text{ext}} \simeq \frac{MW^2}{(M-1)^2} + \mathcal{O}(r^2), \tag{20a}$$

$$C_{EE}^{\text{ext}} \simeq -C_{II}^{\text{ext}} \simeq \frac{W}{M-1} + \mathcal{O}(r^2). \tag{20b}$$

meaning that the external correlations grow linearly with the coupling, but quadratically with the signal-to-noise ratio: a small increase in coupling needs to be followed by a larger increase in signal intensity in order to recover the previous tuning. As a result, the coupling has a larger impact on tuning than the signal-to-noise ratio, an effect that can be measured in the full spiking network.

Finally, we see that between-group correlations also tend to zero as the limit $M \to +\infty$ is taken, since in that case, the input that a module receives from all others becomes just white noise. A finite number of clusters (or finite connectivity among them) is thus required for tuning.

I. Clustering optimisation

Optimization of clustering for a fully connected network can be done by minimizing a loss function that depends on the correlations. A simple possibility is to employ minimum squares,

$$\mathcal{L}^{an}[C; p] = \left(1 - C_{EI}^{\text{int}}\right)^2 + \left(C_{EE}^{\text{ext}}\right)^2 + \left(C_{EI}^{\text{ext}}\right)^2 + \left(C_{II}^{\text{ext}}\right)^2. \tag{21}$$

The solution and associated optimal correlations are shown in N. There are several key remarks following from this figure:

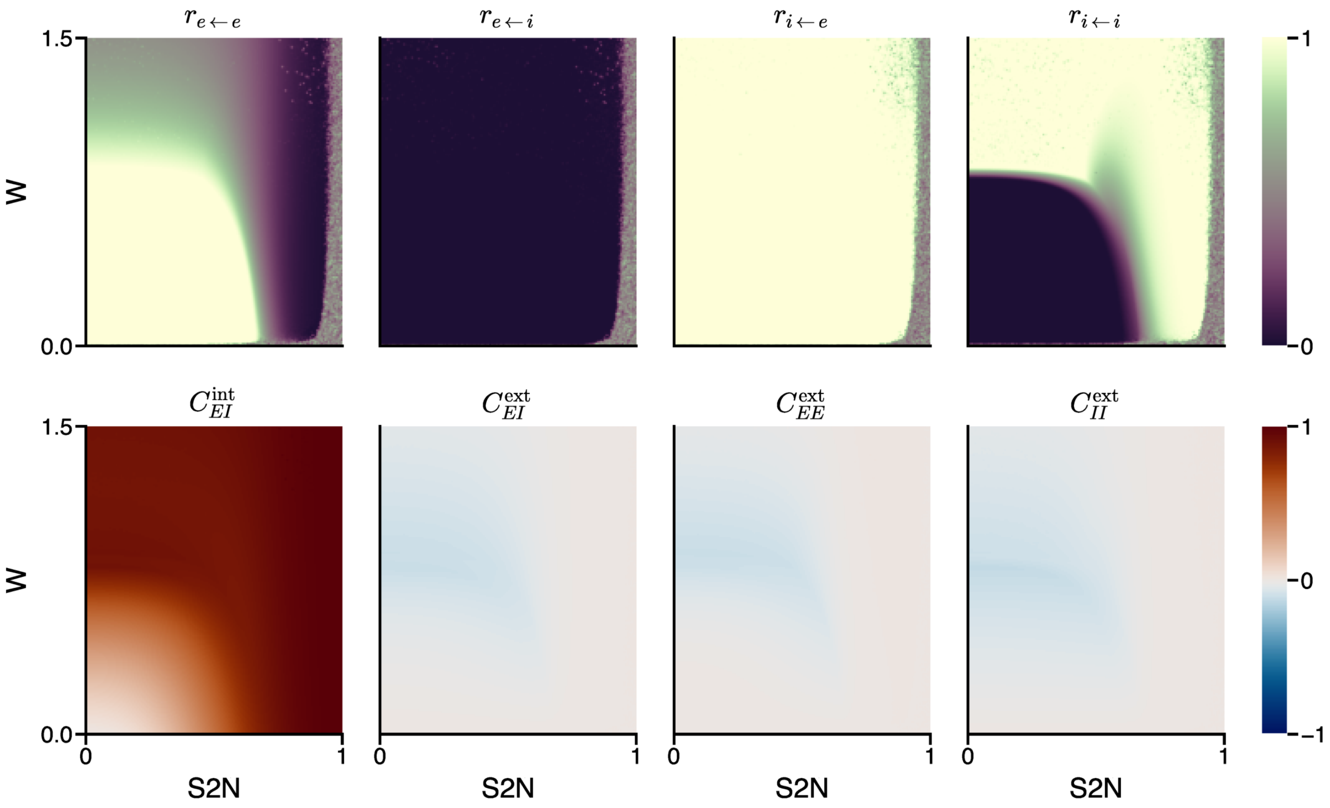

FIG. N. **Optimical clustering for the analytical results.** The selected values of the clustering lead to large decorrelations between different groups. Large correlations in-group can be achieved for high values of recurrence and external noise. In this case, highly clustered excitation and homogeneous inhibition are recovered.

1. Even for very large clustering and extremely low signal-to-noise ratio, the clustering is able to provide an in-group correlation close to unity combined with low between-group correlations, thus ensuring co-tuning.

2. Inhibition to excitation never clusters. Inhibitory neurons act over excitatory individuals regardless of their cluster.

3. Excitatory connections are clustered. In particular, excitatory connections always project to inhibitory neurons in their own cluster but not to other ones. Excitatory-to-excitatory connectivity is also strongly clustered, except for large coupling.

4. Inhibition controls excitation for large W. If one keeps highly clustered excitation and increases the coupling, the dynamics of single modules become unstable at a critical value $W_c(r)$. However, the network can remain stable if the excitatory clustering is reduced and the amount of inhibition in the group increases, which can be accomplished by increasing r_{II}.

5. When the signal-to-noise ratio is close to one, clustering becomes mostly irrelevant, since the system is driven by the external input, which allows co-tuning easily.

Notice that the optimization algorithm automatically finds solutions where the equations are well-defined –i.e., where the system reaches a stationary state– thus selecting to increase the inhibitory clustering when W goes over the instability threshold (Fig N).

Therefore, the analytical approach is able to find a good candidate for optimal clustering depending on the network dynamics. Although it cannot be directly applied to the spiking network, which is able to display richer dynamics, it tells us that, as a rule of thumb, excitatory clustering should be as high as possible while avoiding crossing an instability threshold. If this happens, inhibition needs to be increased.

J. The inferred connectivity structure encourages competition between assemblies

The distribution of optimal assembly strengths we identify (Fig Oa), consists in very strong excitatory assemblies and much weaker inhibitory assemblies, leading to strong excitatory connections among neurons of the same input group and more spread-out inhibitory connections that also target neurons from other groups.

In our network, this global inhibition setting promotes competition between different assemblies which generates the desired correlation structure in the population activity. Essentially, when a group receives an external input, the strong excitatory connections within the group (projecting to both E and I in-group neurons) will lead to high activity of all the neurons (both E and I) in the group. However, unlike the E connections, which largely target other neurons inside the group, the I connections (targeting both E and I neurons) are mostly directed toward the neurons of other groups, which means that the high inhibitory activity inside the group would lead to the suppression of the activity (in both E and I neurons) of other groups.

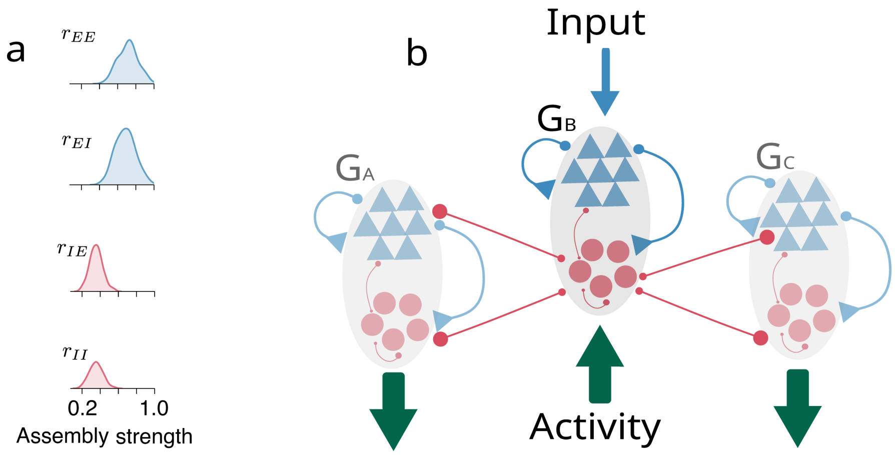

FIG. O. **a** The posterior distrubution of assembly strengths. It promotes very localized excitatory and relatively broad inhibitory connectivity. **b** A diagrammatic representation of how the inferred connectivity is tuned for decorrelating the activities of different groups

K. Tables of parameters

We mostly used the neuron model parameters from the original inhibitory STDP paper [11].

Network Model		
Symbol	Description	Value
N	Number of neurons	1000
N_E	Number of E neurons	800
N_I	Number of I neurons	200
M	Number of input groups	8
g_{leak}	Leak conductance	10 nS
V_{rest}	Resting potential	-60 mV
V_{reset}	Reset potential	-60 mV
V_{th}	Spiking threshold	-50 mV
V_E	Excitatory reversal potential	0 mV
V_I	Inhibitory reversal potential	-80 mV
C_m	Membrane capacitance	200 pF
τ_{ref}	Absolute refractory period	5 ms
τ_E	Decay time constant of E conductance	5 ms
τ_I	Decay time constant of I conductance	10 ms
$\overline{g_E}$	E weight scaling constant	1.4 nS
$\overline{g_I}$	I weight scaling constant	3.5 nS

TABLE A. The parameters used in all simulations (unless otherwise specified) for the network and neuron models

Plasticity Rules		
Symbol	Description	Value
τ_1^{estdp}	Slow eSTDP timescale	50 ms
τ_2^{estdp}	Fast eSTDP timescale	10 ms
η_E	eSTDP learning rate	0.0025
A_{LTP}	Long term potentiation amplitude	1.0
A_{LTD}	Long term depression amplitude	0.2
τ^{istdp}	iSTDP timescale	10 ms
η_I	iSTDP learning rate	0.01
ρ_0	iSTDP target firing rate	3 Hz
η_N	Normalization learning rate	0.003
W_{target}^E	Excitatory normalization target	5.0
W_{target}^I	Inhibitory normalization target	5.0

TABLE B. The parameters used in all simulations (unless otherwise specified) for the plasticity rules

ABC Optimization		
Symbol	Description	Value
α	weight of in-group correlation	0.1
β	weight of between-group correlations	0.3

TABLE C. The parameters used for the loss of the ABC

[1] F. Lagzi and A. L. Fairhall, Emergence of co-tuning in inhibitory neurons as a network phenomenon mediated by randomness, correlations, and homeostatic plasticity, Science Advances **10**, eadi4350 (2024), https://www.science.org/doi/pdf/10.1126/sciadv.adi4350.
[2] S. Deneve and C. Machens, Efficient codes and balanced networks, Nature Neuroscience **19**, 375 (2016).
[3] J. Gjorgjieva, C. Clopath, J. Audet, and J.-P. Pfister, A triplet spike-timing–dependent plasticity model generalizes the bienenstock–cooper–munro rule to higher-order spatiotemporal correlations, Proceedings of the National Academy of Sciences **108**, 19383 (2011), https://www.pnas.org/content/108/48/19383.full.pdf.
[4] A. Schulz, C. Miehl, I. Berry, Michael J, and J. Gjorgjieva, The generation of cortical novelty responses through inhibitory plasticity, eLife **10**, e65309 (2021).
[5] N. Caporale and Y. Dan, Spike timing–dependent plasticity: A hebbian learning rule, Annual review of neuroscience **31**, 25 (2008).
[6] S. Eckmann, E. J. Young, and J. Gjorgjieva, Synapse-type-specific competitive hebbian learning forms functional recurrent networks, Proceedings of the National Academy of Sciences **121**, e2305326121 (2024).
[7] A. Litwin-Kumar and B. Doiron, Formation and maintenance of neuronal assemblies through synaptic plasticity, Nature communications **5**, 5319 (2014).
[8] C. Clopath, T. P. Vogels, R. C. Froemke, and H. Sprekeler, Receptive field formation by interacting excitatory and inhibitory synaptic plasticity, bioRxiv 10.1101/066589 (2016), https://www.biorxiv.org/content/early/2016/07/29/066589.full.pdf.
[9] One could argue that external noise should be interpreted as Stratonovich and internal as Itô. Since both noises are additive, this difference is not so relevant, and we treat both noises as Itô for simplicity.
[10] C. Gardiner, *Stochastic Methods: A Handbook for the Natural and Social Sciences*, Springer Series in Synergetics (Springer, 2009).
[11] T. P. Vogels, H. Sprekeler, F. Zenke, C. Clopath, and W. Gerstner, Inhibitory plasticity balances excitation and inhibition in sensory pathways and memory networks, Science **334**, 1569 (2011), https://science.sciencemag.org/content/334/6062/1569.full.pdf.
